# Molecular Evolution of Phosphoprotein Phosphatases in *Drosophila*

**DOI:** 10.1371/journal.pone.0022218

**Published:** 2011-07-15

**Authors:** Márton Miskei, Csaba Ádám, László Kovács, Zsolt Karányi, Viktor Dombrádi

**Affiliations:** 1 Centre for Agricultural and Applied Economic Sciences, Institute of Horticultural Sciences, Department of Plant Biotechnology, University of Debrecen, Debrecen, Hungary; 2 Department of Medical Chemistry, Research Center for Molecular Medicine, Medical and Health Science Center, University of Debrecen, Debrecen, Hungary; 3 First Department of Medicine, Faculty of Medicine, University of Debrecen, Debrecen, Hungary; Texas A&M University, United States of America

## Abstract

Phosphoprotein phosphatases (PPP), these ancient and important regulatory enzymes are present in all eukaryotic organisms. Based on the genome sequences of 12 *Drosophila* species we traced the evolution of the PPP catalytic subunits and noted a substantial expansion of the gene family. We concluded that the 18–22 PPP genes of *Drosophilidae* were generated from a core set of 8 indispensable phosphatases that are present in most of the insects. Retropositons followed by tandem gene duplications extended the phosphatase repertoire, and sporadic gene losses contributed to the species specific variations in the PPP complement. During the course of these studies we identified 5, up till now uncharacterized phosphatase retrogenes: *PpY+*, *PpD5+*, *PpD6+*, *Pp4+*, and *Pp6+* which are found only in some ancient *Drosophila*. We demonstrated that all of these new PPP genes exhibit a distinct male specific expression. In addition to the changes in gene numbers, the intron-exon structure and the chromosomal localization of several PPP genes was also altered during evolution. The G−C content of the coding regions decreased when a gene moved into the heterochromatic region of chromosome Y. Thus the PPP enzymes exemplify the various types of dynamic rearrangements that accompany the molecular evolution of a gene family in *Drosophilidae*.

## Introduction

Protein phosphorylation is a frequent postsynthetic modification operating in all eukaryotic organisms. The protein kinase enzymes that are responsible for the phosphorylation of Ser, Thr and Tyr residues of proteins evolved from a single ancestor. From the point of view of regulation it is obvious that the kinases must cooperate with protein phosphatases in order to ensure the reversibility of the process. The significance of these two competing enzyme families is equivalent in propelling the phosphorylation-dephosphorylation based regulatory cycles; however the phosphatases are second to the kinases in two respects. (i) According to the reaction mechanisms kinases should act first by modifying the side-chains in the nascent polypeptides. (ii) Phosphatases must have evolved after the kinases, since in the absence of the phosphoprotein substrate they would have had no useful function. Consequently, protein phosphatases were recruited from different fields. Some of the already existing hydrolytic enzymes adopted their catalytic pockets to accommodate the new substrates and slowly acquired more and more specificity. The so-called PhosphoProtein Phosphatase (PPP) enzymes developed from the bacterial diadenosine tetraphosphatases [1]. They are probably the most ancient protein phosphatases as the representatives of the family can be found in some prokaryotes [2], and are present in all eukaryotes [3]. They specifically dephosphorylate the Ser and Thr residues of proteins in a bicentral metal ion assisted hydrolytic reaction [4], and play fundamental roles in regulating a diverse array of cellular functions [5].

**Figure 1:**
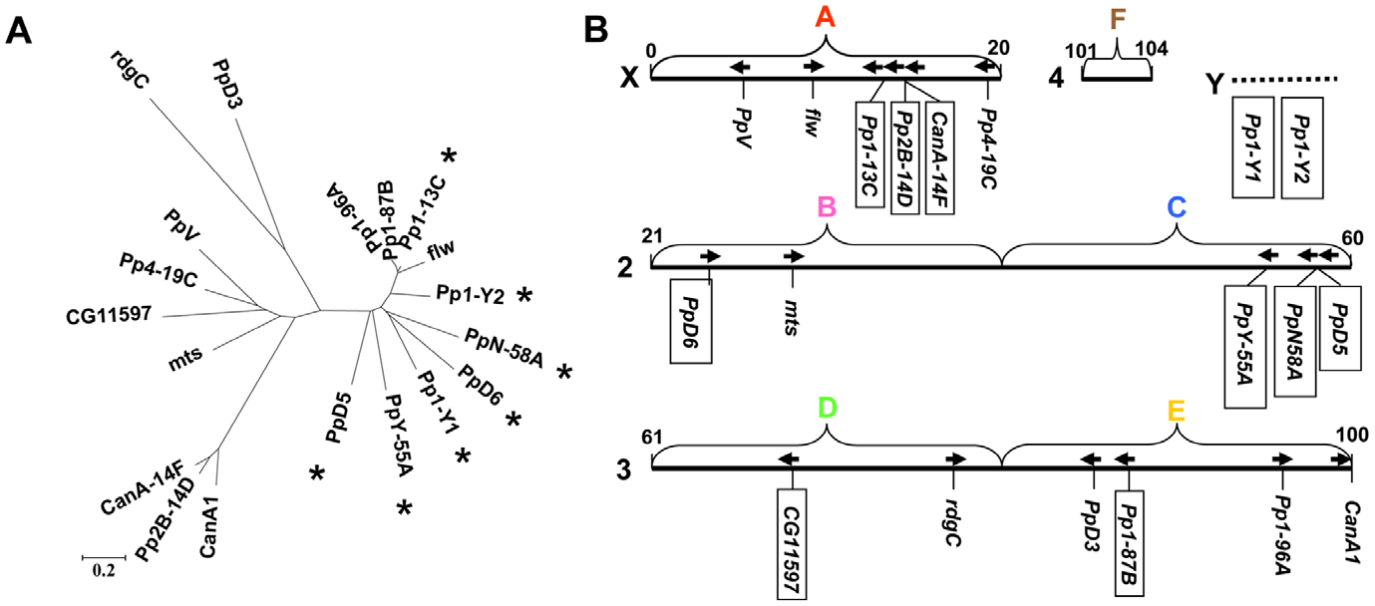
Phosphoprotein phosphatase (PPP) catalytic subunits of *D. melanogaster*. (**A**) Phylogenetic relationship of the amino acid sequences of 19 phosphatases. The bar indicates 0.2 amino acid substitutions per site, * labels male specific expression. (**B**) Chromosomal localization of the PPP genes. Chromosomes X, 2, 3, and 4 are depicted as solid bars with the numbering of the first and the last divisions. Chromosome Y is represented by an unproportional broken bar. The capital letters above the chromosomes label the chromosome arms termed the Muller elements. Although chromosome numbers vary in different *Drosophila* species the Muller elements are common to all species and can be conveniently used for the comparison of a gene's global location. The directions of the ORFs are indicated by arrows (left to right corresponds to 5′ to 3′ in the upper strand). The genes that have no introns in their coding regions are boxed.

*D. melanogaster* is a well-established model organism of molecular genetics. A recent survey of the FlyBase ([6], http://flybase.bio.indiana.edu/) identified 19 genes coding for PPP catalytic subunits in this organism (**Figure 1A**). According to their primary structures the PPP enzymes can be divided into 5 subgroups [5]. (i) The type 1 or PPP1 subfamily includes 4 of the classical PP1 paralogs that were named according to their chromosomal locations (**Figure 1B**). *Pp1-13C*, *Pp1-87B*, and *Pp1-96A* are the *Drosophila* orthologs of the mammalian PPP1 alpha isoform, while *Pp1-9C* corresponds to mammalian PPP1 beta/delta [7]. In addition, there are 6 novel members: *PpY-55A*, *PpN-56A*, *PpD5*, *PpD6*, *Pp1-Y1*, and *Pp1-Y2*, which are *Drosophila* specific intronless phosphatases with male biased expression [8]. (ii) The calcineurin/Pp2B/PPP3 Ca-regulated protein phosphatases are represented by 3 closely related isoforms (**Figure 1A**). *CanA1* [9] is a huge gene with 12 introns, while *Pp2B-14D* [10] and *CanA-14F* have no introns in their coding regions. (iii) In *D. melanogaster* there are 4 type 2 phosphatases. The first member of the subfamily, *Pp2A* is also called *microtubule star* (*mts*) because its mutation resulted in star like arrangement of microtubules [11], *Pp4-19C* was originally described as *PPX* [12], and *PpV* is the *Drosophila* ortholog of budding yeast *SIT4* and mammalian *Pp6* [13]. *CG11597* was identified by genome database mining and was termed as *Pp4-like* by Morrison et al. [14]. Since we found a phosphatase that is even more similar to *Pp4* (see **Table 1**) we prefer to use the *CG11597* annotation throughout this manuscript. (iv) *PpD3* still keeps the name of a PCR product that lead to its discovery [15]. It is the ortholog of mammalian PPP5/*Pp5* [16]. (v) *Retinal degeneration C (rdgC)* was discovered as a Ca-calmodulin regulated protein phosphatase protecting retina from light-induced degeneration [17]. It is similar to human PPP7/*Pp7* isoenzymes termed PPEF-1 and PPEF-2 in as much as it has a C-terminal EF-hand containing regulatory domain.

**Table 1:** Localization of PPP genes in Muller elements of 12 *Drosophila* species.

Classification^a^	Gene name, synonym^b^/Species^c^	Dmel	Dsim	Dsec	Dyak	Dere	Dana	Dpse	Dper	Dwil	Dmoj	Dvir	Dgri
Type 1or PPP1	***Pp1α-96A, Pp1-96A***	**E**	**E**	**E**	**E**	**E**	**E**	**E**	**E**	**E**	**E**	**E**	**E**
	*Pp1-87B*	***E***	***E***	***E***	***E***	***E***	***E***	***E***	***E***	***E***	***E***	***E***	***E***
	*Pp1-13C*	***A***	***A***	***A***	***A***	***A***	***A***	***A***	***A***	***A***	***A***	***A***	***A***
	***flw, Pp1-9C***	**A**	**A**	**A**	**A**	**A**	**A**	**A**	**A**	**A**	**A**	**A**	**A**
	*Pp1-Y1*	***Y*** h	***Y*** g	***Y*** e	***Y*** g	***Y*** h	***Y***	***C***	***C***	***C***	**x**	***C***	***C***
	*Pp1-Y2*	***Y*** f	***Y*** g	***Y*** h	***Y***	***Y***	***Y*** i	***C***	***C***	***C***	***C***	***C***	***C***
	*PpD5*	***C***	***C*** g	***C***	***C***	***C***	***C***	***C***	***C*** h	**x**	***C***	***C***	**x**
	*PpD5+*	**x**	**x**	**x**	**x**	**x**	**x**	**D**	**D**	**x**	***D***	***D***	***D***
	*PpD6*	***B***	***B***	***B***	***B***	***B*** h	***Y*** f ***^,^*** i	***C*** h	***C***	***C***	***C***	***C***	***C***
	*PpD6+*	**x**	**x**	**x**	**x**	**x**	**x**	***C***	***C*** h	***C***	**x**	***C***	**x**
	*PpN58A*	***C***	***C***	***C***	***C***	***C***	***C***	***C***	***C***	***C***	***C***	***C***	***C***
	*PpY-55A*	***C***	***C***	***C***	***C***	***C***	***C***	***A***	***A*** h	**C**	**C**	**C**	**x**
	*PpY+*							***A***	***A***				
Calcineurinor Pp2Bor PPP3	***CanA1***	**E**	**E**	**E**	**E**	**E**	**E**	**E**	**E**	**E**	**E**	**E**	**E**
	*Pp2B-14D (14E)* d	***A***	***A***	***A***	***A***	***A***	***A***	***A***	***A***	***A***	***A***	***A***	***A***
	*CanA-14F*	***A*** h	***A*** e	***A*** h	***A***	***A***	***A***	***A***	***A*** h	***A***	***A***	***A***	***A***
Type 2or PPP2-4-6	***mts, Pp2A***	**B**	**B**	**B**	**B**	**B**	**B**	**B**	**B**	**B**	**B**	**B**	**B**
	***Pp4-19C*** * (19D)* d	**A**	**A**	**A**	**A**	**A**	**A**	**A**	**A**	**A**	**A**	**A**	**A**
	*Pp4+*							***B***	***B***				
	***PpV, Pp6***	**A**	**A**	**A**	**A**	**A**	**A**	**A**	**A**	**A**	**A**	**A**	**A**
	*Pp6+*										***E***	***E***	***E***
	*CG11597*	***D***	***D***	***D***	***D***	***D***	***D***						
PPP5	***PpD3, Pp5***	**E**	**E**	**E**	**E**	**E**	**E**	**E**	**E**	**E**	**E**	**E**	**E**
PPP7	***rdgC, Pp7***	**D**	**D**	**D**	**D**	**D**	**D**	**D**	**D**	**D**	**D**	**D**	**D**
PPP	Total number	**19**	**19**	**19**	**19**	**19**	**19**	**22**	**22**	**18**	**19**	**21**	**18**

a According to Cohen [65].

b The gene names are given according to Flybase (http://flybase.org/) and are followed by frequently used synonyms. The names of the genes that constitute the minimal PPP toolkit are written in bold face.

c Species names are abbreviated as follows: Dmel: *D. melanogaster*, Dsim: *D. simulans*, Dsec: *D. sechellia*, Dyak: D. *yakuba*, Dere: D. *erecta*, Dana: *D. ananassae*, Dpse: *D. pseudoobscura*, Dper: *D. persimilis*, Dwil: *D. willistoni*, Dmoj: *D. mojevensis*, Dvir: *D. virilis*, Dgri: *D. grimshawi*.

d The correct chromosomal localization of a gene is given in parentheses if different from the position inferred from the gene name.

These gene sequences were

e identified,

f revised, or

g confirmed by PCR and DNA sequencing.

h The gene size was confirmed by PCR.

i The gene localization was corrected on the bases of genome environment.

x stands for a lost/missing gene.

The localization of intronless genes is highlighted in underlined italics.

Since *D. melanogaster* has the largest number of PPP genes in the animal kingdom we became interested in the origins of this family of regulatory enzyme. The gene structures (**Figure 1B**), and the expression patterns (**Figure 1A**) suggest that many of the PPP enzymes of *D. melanogaster* are functional retrogenes. However, the analysis of a well characterized species alone would have been fruitless from the point of evolutionary studies. The publication of the genome *sequences* of 12 *Drosophila* species [18] opened a new avenue for the comparative analysis of gene families [19]. Obviously the expansion of a gene family requires the origination of new genes. According to recent estimates about 17 genes did duplicate in the *Drosophilidae* within 1 million years [19], while 5–11 genes originated in the *melanogaster* subgroup within the same period of time [20]. The majority of the new genes is generated by tandem or dispersed duplications; in addition retropositions, formation of chimeric genes, and *de novo* gene origination from noncoding sequences contribute to the extension of the gene repertoire [20]. However, the gene number in a given family is also affected by deletion or degradation of some genes; a dynamic balance between gains and losses is a characteristic feature of genome evolution [19]. By comparing the nucleotide and the amino acid sequences in *Drosophilidae* we ventured on disclosing the evolution of the relatively small and conservative gene family of phosphoprotein phosphatases. We also analyzed the databases of 8 completely sequenced insects (summarized in FlyBase, [6]) in order to reveal the origins of the novel *Drosophila* specific PPP family members. In our study we found that the rate of PPP gene gains exceeded that of gene losses and noted dramatic rearrangements affecting the PPP gene family in the fly genomes.

## Methods

*Drosophila melanogaster* Canton S strain was obtained from the Szeged Drosophila Stock Center (http://expbio.bio.u-szeged.hu/fly/index.php) that has been moved recently to Kyoto (http://kyotofly.kit.jp/cgi-bin/stocks/index.cgi). *Drosophila pseudoobscura*, *Drosophila virilis*, *Drosophila persimilis*, and *Drosophila willistoni* were from the Bloomington Drosophila Stock Center (http://flystocks.bio.indiana.edu/News/sequenced.htm). The fruit flies were cultivated on standard corn food at 25°C.

Genomic DNA was extracted from single flies according to the protocol of Gloor and Engels [21]. Total RNA was isolated with the Trizol Reagent (Invitrogen) and was treated with RNase-free DNase (Promega). Phosphatase specific oligonucleotide primers were designed with the Oligo Explorer 1.1.0 primer selection software developed by Teemu Kuulasmaa (http://oligo-explorer.software.informer.com). Total RNA was reverse-transcribed with M-MLV Reverse Transcriptase (Promega) and oligo-dT primer. Genomic DNA and cDNA were amplified by PCR, the reaction conditions and primer sequences are summarized in **Table S1**. If a PPP gene sequence was missing from the databases primers were designed based on the homologous gene sequences of the most closely related *Drosophila* species (**Table S1B**). The PCR products were subjected to electrophoresis in 1% agarose gels, stained with ethidium bromide, and visualized in a FluorChem FC2 Imaging System (Alpha Innotech). Some of the amplicons were purified by a Wizard SV gel and PCR clean-up kit (Promega) and were sequenced by the chain termination method.

Sequence data were collected from the FlyBase (http://flybase.org/), the UCSC Genome Bioinformatics (http://genome.ucsc.edu/) and the NCBI (http://www.ncbi.nlm.nih.gov/), databases. Initially 19 known *D. melanogaster* PPP protein sequences (Figure 1) were used as queries in blastp for homology search [22]. ORFs in the UCSC Genome Bioinformatics DNA sequences were predicted with pDRAW32 (http://www.acaclone.com/), FGENESH and FGENESH+ [23] programs. Hits of the first round were used as queries in a second blastp search in order to confirm, and extend the sequence collection. In both searches a strict limitation of the expect value (E<1×10^−40^) was used [24]. New hypothetical protein sequences were also examined with the SMART software (a Simple Modular Architecture Research Tool) in order to identify their domain structures (http://smart.embl-heidelberg.de/). Our initial protein assignation was accepted only if a typical PPPs catalytic domain was revealed by SMART. Obvious mistakes of the databases (frame shifts, premature stops, insertions and deletions) resulting in an aberrant protein that was not compatible with the conserved primary structures of the closely related PPP enzymes were corrected manually with the help of pDRAW32 (Table S2). Homologous protein sequences were compared with the ClustalW program [25]. Known PPPs were identified by the abridged species name followed by the corresponding *D. melanogaster* phosphatase name as given in FlyBase (**Table 1**). If a phosphatase gene coded for more than one isoforms always the longest protein sequence was used for the analysis.

Protein sequences were compared with the pDRAW32 and the BioEdit [26] softwares. Phylogenetic and molecular evolutionary calculations were conducted with MEGA version 4.0 [27], [28] using Neighbor-Joining method in the Dayhoff matrix substitution model [29], [30]. Bootstrap tests were performed with 500 replications [31]. Orthologous relationships were deduced from the tree topology and were confirmed by microsyntheny analysis. The complex type 1 PPP subfamily was also analyzed by the multidimensional scaling (MDS) method [32], [33] with the SAS for Windows 8.2, PROC MDS procedure (Cary NC, USA, SAS Institute Inc.).

Gene localizations in the Muller elements were taken from FlyBase [6]. Based on their genome environments the chromosomal localizations of *Pp1-Y2* and *PpD6* genes in *D. ananassae* were corrected (**Table 1**). Genetic rearrangements were examined by dot plots prepared with pDRAW32. The G−C percentage of the coding regions was calculated with OligoExplorer (http://oligo-explorer.software.informer.com). The dn/ds values for the PPP catalytic domain coding sequences were calculated and analyzed as described earlier [8].

## Results

### The basic PPP toolkit of insects

We used *D. melanogaster* as a gold standard for the comparison of the PPP catalytic subunits in 12 *Drosophila* species. **Table 1** demonstrates that with a few exceptions all of the PPP genes of *D. melanogaster* are also present in the genomes of the other 11 members of *Drosophilidae*, total PPP numbers fluctuate between 18 and 22 per species. The minimal PPP toolkit was predicted from **Table 2** comprising 7–8 enzymes that are present in most of the sequenced insect species.

**Table 2:** Identification of PPP genes in sequenced insect genomes.

Gene, Class^a^	*Culex* *quinquefasciatus*	*Aedes* *aegypti*	*Anopheles* *gambiae*	*Tribolium* *castaneum*	*Apis* *mellifera*	*Nasonia* *vitripennis*	*Acyrthosiphon* *pisum*	*Pediculus* *humanus corporis*
Abbreviations	*Cqui*	*Aaeg*	*Agam*	*Tcas*	*Amel*	*Nvit*	*Apis*	*Phum*
***flw*** **, PPP1**	XP_001843526	XP_001663366	XP_312797	XP_966417	XP_623273	XP_001604472	XP_001944422	EEB19394
***Pp1α-96A*** **, PPP1**	XP_001849462	XP_001653770	XP_309483	XP_001813974	XM_392943	XP_001602738	XM_001945867	
***mts*** **, PPP2**	XP_001863301	XP_001663281	XP_319345	XP_973546	XP_623105	XP_001602506	NP_001119644	EEB13988
***Pp4-19C*** **, PPP4**	XP_001843269	XP_001648308	XP_310323	XR_043119	XM_624666	XP_001606225	XP_001950610	EEB16959
***PpV*** **, PPP6**	XP_001866724	XP_001648846	XP_311859	XP_967314	XP_394400	XP_001603727	XP_001951846	EEB14281
***CanA1*** **, PPP3**				XP_968705	XP_394519	XP_001602102 (A)^c^XP_001603722 (B)^c^	XP_001945831	EEB19897
***Pp2B*** **, PPP3**	XP_001868819	XP_001653535	Revised sequence b					
***PpD3*** **, PPP5**	XP_001850926	XP_001650298	XP_313034	XP_971407	XP_624242	XP_001603324	XP_001948640	EEB13025
***rdgC*** **, PPP7**	XP_001844975	XP_001663541	XP_317894					EEB16582
**Total, PPP**	**8**	**8**	**8**	**7**	**7**	**8**	**7**	**7**

a Genes are termed as the *D. melanogaster* orthologs, PPP classification is given according to Cohen [65].

b The revised protein sequence can be found at http://www.medchem.dote.hu/hu/node/1127.

c The two Nvit_CanA1 sequences have been arbitrarily differentiated as A and B.

PPP genes that contain no introns in their coding regions are underlined. The two types of PPP3 are separated by a broken line.

**Figure 2:**
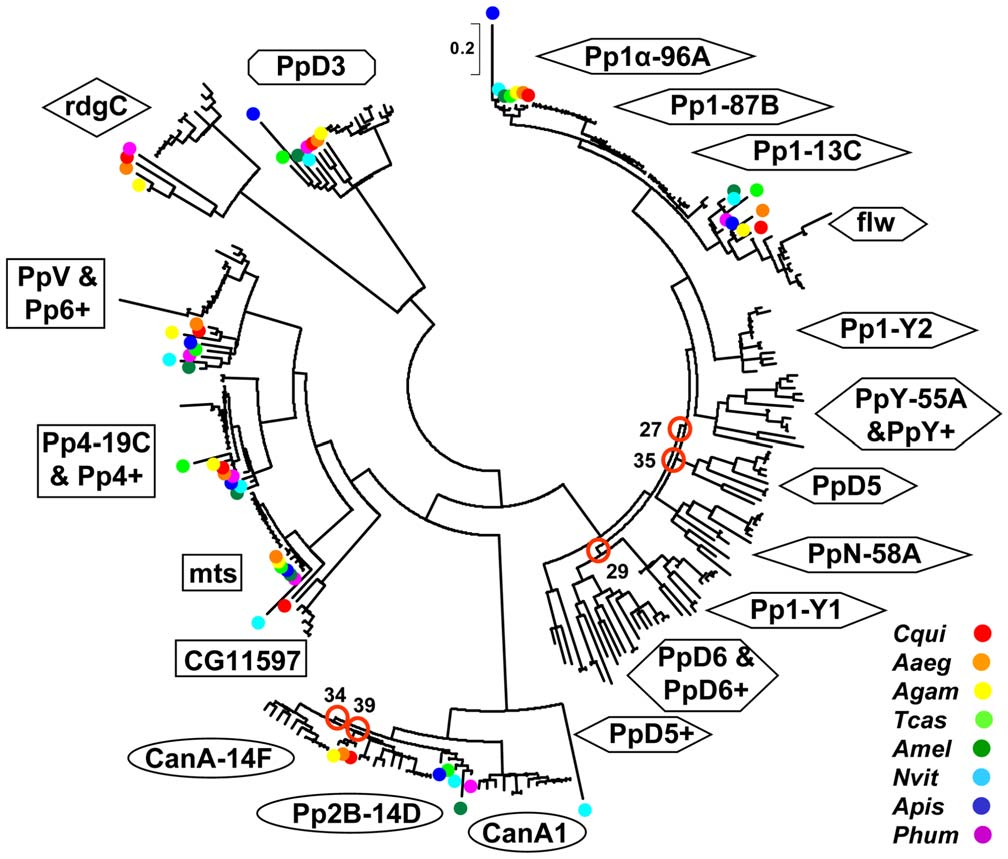
Phylogeny of PPP catalytic subunits in insects. The phylogeny of 287 insect PPP catalytic subunits is presented. Enzyme subfamilies are labeled in line with the *D. melanogaster* nomenclature (see **Table 1**). The color codes for the non-*Drosophila* species are given in the lower right corner, species abbreviations are listed in **Table 2**. The scale indicates 0.2 amino acid substitutions per site in the primary structure. Uncertain branching points are circled and labeled with the appropriate bootstrap value. The following 8 short, partial polypeptide sequences were excluded from the comparisons: Dsim flw, Dana *Pp1-Y1*, Dsec *Pp1-Y2*, Dwil *PpN58A*, Dyak *Pp2B-14D*, Dvir *Pp2B-14D*, Dsim *CanA-14F*, Dsim *rdgC*.

The structural relationships of the insect PPP protein sequences are depicted in **Figure 2** and **Figure S1** contains the identification of each protein as well as the bootstrap values of the family tree. Both of the figures show that 7 of the 8 common insect phosphatases fall into 7 well-defined, distinct groups in the family tree demonstrating a conserved structure and suggesting a conserved function for these enzymes in *Drosophila* and other insects. The phosphatases of the non-*Drosophila* species (that are labeled with colored dots) form sub-branches that are well separated from the *Drosophila* proteins supporting a common origin but independent evolution.

**Figure 3:**
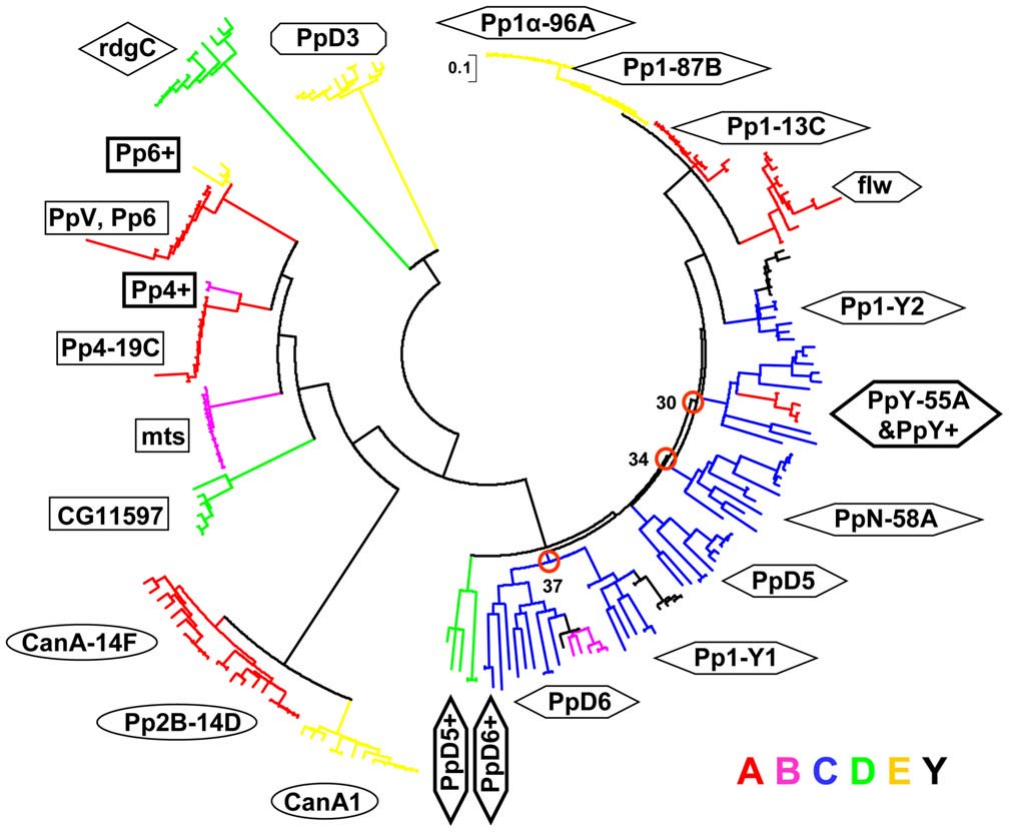
Phylogeny of PPP catalytic subunits in *Drosophilidae*. The phylogenetic analysis of 227 *Drosophila* PPP catalytic subunits is presented as in **Figure 2**. The color of the branches indicates the chromosomal localization of the corresponding genes, the color code of the Muller elements is given in the lower right corner. The names of the 5 novel PPP enzymes that were identified in the present study are labeled by a+suffix and are surrounded by a heavy border. The scale indicates 0.1 amino acid substitutions per site in the primary structure.

The only exception to the rule is the group of PPP3/calcineurin (*CanA1/Pp2B*) phosphatases. First of all, the B variant of *N. vitripennis CanA1* is an outlying member of the subfamily (**Figure 2**). We assume that it was derived from the more conserved A variant by gene duplication, and its sequence diverged significantly due to its functional redundancy. **Figure 2** shows that 5 of the non-*Drosophila* PPP3 enzymes are similar to *Drosophila CanA1*. Typical *CanA1* genes contain introns in their coding regions (**Tables 1** **,** **2**). In contrast, the PPP3 phosphatases of *C. quinquefasciatus*, *A. egypty*, and *A. gambiae* lay closer to *Drosophila Pp2B-14D* enzymes according to their protein (**Figure 2**) and gene (**Tables 1** **,** **2**) structures. These intronless genes testify the duplication of the ancient intron containing *CanA1* gene in *Diptera*. Consequently, we consider *CanA1* as the ancestor of all insect calcineurin phosphatases that was retained in *Drosophilidae* but was lost in other *Diptera*. The presence of 3 independently evolving non-*Drosophila Pp2B-14* enzymes created some uncertainty in the family tree construction (**Figure 2**) that was eliminated when the analysis was focused on the 12 *Drosophila* species (**Figure 3**).

**Figure 2** also demonstrates that several groups of *Drosophila* phosphatases have no orthologs in other insects. Strikingly, in addition to the standard phosphatase repertoire, the members of *Drosophilidae* acquired no less than 15 new, dynamically changing PPP family members (**Table 1**). These new phosphatases are emphasized in **Figure 3**, while the detailed family trees of the 12 *Drosophila* species as well as that of the PPP protein sequences are given in **Figures S2A** and **S2B**. The obvious question arises how the new phosphatases evolved; what was the mechanism of the gain and occasional loss of PPP genes?

### Gain of PPP genes in *Drosophilidae*

Duplications are supposed to be the most effective tools extending gene repertoire. Our data support the thesis that both *Pp2B-14D (14E)* and *CanA-14F* are the intronless derivatives of *CanA1* in all *Drosophila* species (**Figure 3** and **Table 1**). The comparison of the phosphatases in insects (**Figure 2**), and especially in 12 *Drosophila* species (**Figure 3**) suggests that an intron containing ancient form of *Pp1-96A* was the parent of the intronless type 1 phosphatases. *Pp1-87B* evolved directly from the *Pp1-96A*. Based on the available data it is more difficult to tell if *Pp1-13C* originated directly from *Pp1-96A*, or indirectly *via Pp1-87B*, however it is sure, that the other intron containing type 1 phosphatase, *flw*, can not be the common ancestor as it is more variable and less closely related (**Figures 3** and **S2D**).

**Figure 4:**
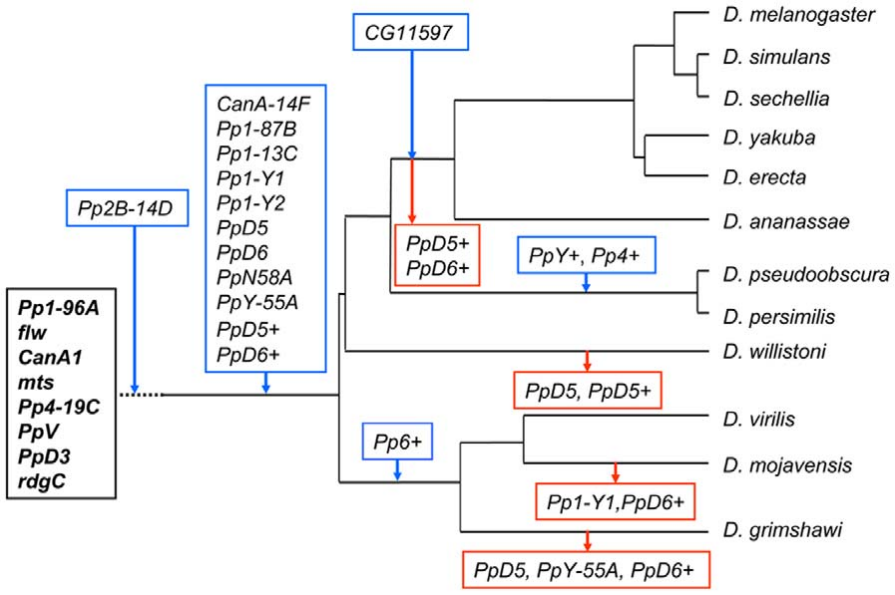
Gain and loss of PPP genes in *Drosophilidae*. The basic PPP set of insects is in a black box. Gained genes are in blue and lost genes are in red boxes. The broken line indicates that the *Pp2B-14D* gene was gained in the *Diptera*. The family tree of the 12 *Drosophila* species with a mutational clock is presented in **Figure S1A**.

In addition to the duplication of classical phosphatase genes, the PPP set of *Drosophilidae* was substantially expanded by the appearance of more retrogenes (**Figure 4**). The novel type 1 phosphatases, i.e. *Pp1-Y1*, *Pp1-Y2*, *PpD5*, *PpD6*, *PpN58A*, and *PpY-55A* that were originally discovered in *D. melanogaster* (**Figure 1**), are present in all of the 12 *Drosophila* species (**Figure 3** and **Table 1**), but are absent from other insects (**Figure 2** and **Table 2**). As a matter of fact, their orthologs cannot be found in any other living organisms, thus they can be classified as *Drosophila* specific phosphatases.

### New PPP genes of *Drosophilidae*

Unexpectedly, retropositions and tandem gene duplications created additional new PPP enzymes that do not exist in *D. melanogaster*. Previously, Koerich et al. [34] have noted that *D. willistoni* and *D. virilis* contained a gene that was similar to *PpD6*, however they did not explore this observation. We considered the finding of two orthologs of a given *D. melanogaster* phosphatase in the same species as a sign of gene duplication. One of these paralogs, which was more similar to the *D. melanogaster* query sequence, was assumed to carry the functions of the known gene, while the less similar one was regarded as a new gene copy. For the assignation of the new members of the family we used the name of the *D. melanogaster* ortholog with a+suffix. Our gene assignation was confirmed by the different chromosomal localization of the two paralogs, and the fact that the old copy preserved its original location (Figure S5). The functional activity of the novel gene copy was proven by the detection of its mRNA transcript (Table 3). Following up this approach we identified 5 up till now uncharacterized phosphatases (**Figure 3** and **Table 1**). According to our sequence comparisons (**Figure S2C**) it is likely that *PpD6+* and *PpD5+* were derived from *PpD6* and *PpD5*, respectively. These two new retrogenes are present in most of the *Drosophila* species with the exception of the melanogaster group (**Table 1**), thus they must have appeared together with the other, better known PPP retrogenes more than 63 million years ago when the *Drosophilidae* separated from other insects (**Figure 4**). The *Pp6+* gene was identified exclusively in the representatives of the ancient *Drosophila* subspecies placing the time of its appearance in the window of 44–63 million years ago (**Figure 4**). A more recent duplication of the *Pp4* gene producing *CG11597* was described earlier [8]. **Table 1** confirms that this gene is restricted to the members of the melanogaster group that bifurcated from the obscura group 56 million years ago (**Figures 4** and **S2A**).

**Table 3:** Expression of the new PPP genes in *Drosophila* imagos.

Gene name^a^/Species	Dmel		Dana		Dpse		Dwil		Dvir	
Sex^b^	M	F	M	F	M	F	M	F	M	F
*PpD5* c	**+**	**−**	**+**	**−**	**+**	**−**			**+**	**−**
***PpD5+***					**+**	**−**			**+**	**−**
*PpD6* c	**+**	**−**	**+**	**−**	**+**	**−**	**+**	**−**	**+**	**−**
***PpD6+***					**+**	**−**	**+**	**−**	**+**	**−**
*PpY-55A* c	**+**	**−**	**+**	**−**	**+**	**−**	**+**	**−**	**+**	**−**
***PpY+***					**+**	**−**				
*Pp4-19C*	**+**	**+**	**+**	**+**	**+**	**+**	**+**	**+**	**+**	**+**
***Pp4+***					**+**	**(+)/−**				
*PpV, Pp6*	**+**	**+**	**+**	**+**	**+**	**+**	**+**	**+**	**+**	**+**
***Pp6+***									**+**	**−**
*Pp1-13C*	**+**	**−**	**+**	**+/−**	**+**	**+/−**	**+**	**+/−**	**+**	**+/−**

a The gene names are as in Table 1. New PPP genes are in bold.

b Sexes are denoted as F: female, and M: male.

c Adam et al. [8].

Empty fields indicate missing/unidentified genes.

Two of the closely related representatives of the obscura group, *D. pseudoobscura*, and *D. persimilis* gained the group specific *PpY+* and *Pp4+* retrogenes (**Table 1**). The similarity between the *PpY-55A* and *PpY+* sequences (**Figures 3** and **S2B**), as well as the close localization of the two genes in the physical map of Muller element A (**Figure S5A**) suggest that the latter was generated from the former by replicative transposition. Likewise, the sequence analysis of the *Pp4-19C* and *Pp4+* pairs (**Figures 3** and **S2B**) reveals that the retroposition of the intron containing *Pp4-19C* produced the intronless *Pp4+* gene copy.

Although the new PPP genes code for full length proteins that contain the critical residues which are necessary for phosphatase activity, without experimentation it would be rather difficult to predict if they were transcribed, or were degraded to an inactive pseudogene status.

We tested by RT-PCR the expression of the 5 new PPP genes identified in the present study as well as the expression of their parental genes. **Table 3** and **Figure S3** demonstrate that all of the new genes are functionally active retrogenes. Most of them have a strict male-specific expression pattern, the only exception is *Pp4+* that is slightly expressed in females, too. The male-specific transcription of *PpD5*, *PpD6*, and *PpY-55A* genes has been retained by their new paralogs *PpD5+*, *PpD6+*, and *PpY+*. On the other hand, the paralogs of the otherwise unbiased parental *Pp4-19C* and *PpV*, *Pp6* genes acquired sex preference in expression (**Table 3**).

### Loss of PPP genes from *Drosophilidae*

The gene duplications described in the previous sections explain how the basic PPP gene set of insects was amended by new members and how *Drosophila* species gained their sizeable PPP complements. However, the situation is more complicated, since some of the new PPP genes have been lost during the course of evolution (**Table 1**).

The absence of a gene from the database may be attributed to the intrinsic shortcoming of the shotgun sequencing strategy. To overcome this problem we used PCR (**Table S1**) for the amplification and identification of the missing genes. In this way we found 2 of the PPP genes that were not recovered by the genome projects, corrected 2, and confirmed 4 gene sequences (**Tables 1**, **S2**). Still there remained many missing PPP genes that were not recovered by our experimental approach. One can argue that these genes were not identified for technical reasons, as they are in species that have only distant sequenced relatives preventing the construction of appropriate PCR primers. However, it should not escape our attention that the absences do not follow a probabilistic distribution, in fact all of the missing PPP enzymes belong to the novel type 1 retrogenes (**Table 1**).

**Figure 5:**
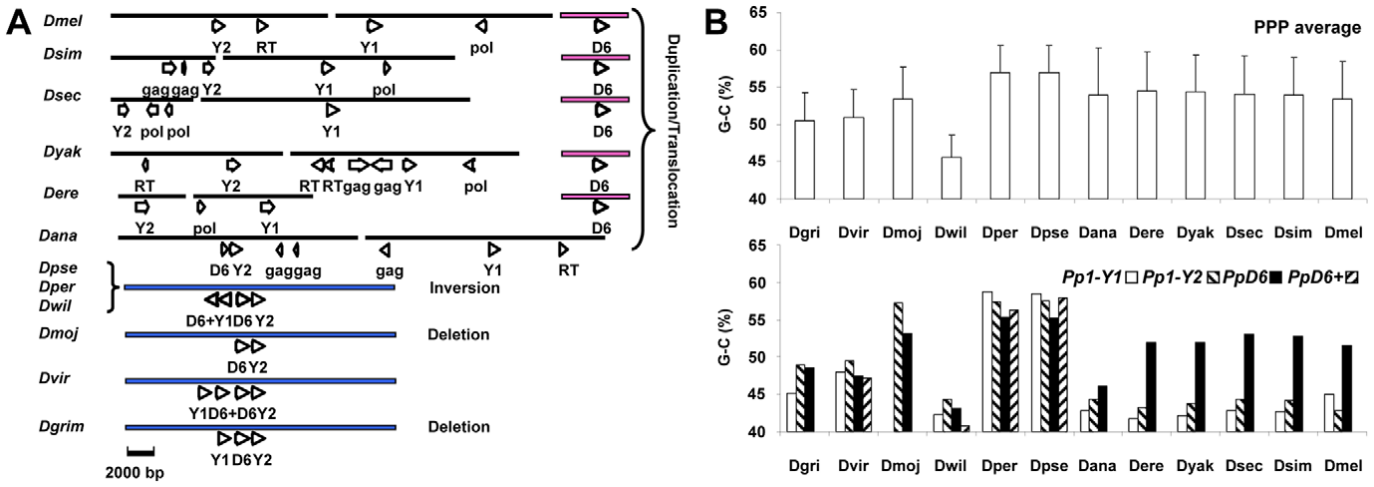
Rearrangements of the *PpD6*, *PpD6+*, *Pp1- Y1*, *Pp1-Y2* gene cluster. (**A**) Chromosomal localizations of *PpD6* (D6), *PpD6+* (D6+), *Pp1- Y1* (Y1), and *Pp1-Y2* (Y2) genes. Chromosome segments are symbolized by horizontal bars, the coloring identifies Muller elements C, B and chromosome Y in blue, pink, and black, respectively. The directions of the coding regions are indicated by arrows, pointing from 5′ to 3′ in the sense strand. In chromosome Y the positions and orientations of inactive viral reverse transcriptase (*RT*), *pol* and *gag* genes are also shown for orientation; here the arrows represent only the relative directions within a short DNA segment. (**B**) The G−C content in the coding regions of the *PpD6*, *PpD6+*, *Pp1- Y*, and *Pp1-Y2* genes. The upper part of the panel shows the average G−C content + SD for all PPP genes of a given species, while the lower part depicts the G−C content of the four selected phosphatases.

*PpD5+* and *PpD6+* were eliminated from the melanogaster group 56–44 million years ago (**Figure 4**). The explanation of the accidental, species specific gene losses requires more consideration (**Figure S4**). The lack of *Pp1-Y1* and *PpD6+* from *D. mojavensis* and the absence of *PpD6+* from *D. grimshawi* have already been reported by Koerich et al. [34], and can be attributed to the rearrangements of an ancient PPP gene cluster (**Figure 5**). The *PpY-55A* gene was not identified in *D. grimshawi* (**Table 1**). We noted that the chromosomal region in Muller element C that is supposed to contain *PpY-55A* between the landmark genes *elk* and *drp13* underwent substantial rearrangements in this species, but we did not find the *PpY-55A* sequence in the expected position(s) either in the vicinity of *elk* or close to *drp13* (**Figure S4A**). Thus, the Hawaiian drosophila lost its *PpY-55A* (or this gene has not been sequenced yet for technical reasons). The deletion of *PpD5+* from *D. willistoni* is supported by the synteny and dot plot analysis of its genomic environment (**Figure S4C, D**). A similar analysis suggests that *PpD5* was deleted/degraded in *D. willistoni* and *D. grimshawi* (**Figure S4E, F**). Thus the investigation of the affected chromosome regions confirms the accidental loss of some redundant type 1 PPP genes (**Figure 4**).

### Relocations and rearrangements of PPP genes in *Drosophilidae*

Besides the gain and loss of genes the changes in the chromosomal localizations and gene structures also contributed to the evolution of the PPP family in *Drosophilidae*. For reference, the localizations of PPP genes in the Muller elements of *D. melanogaster* are shown in **Figure 1B**. Gene movements between Muller elements are outlined in **Table 1** and are reflected by the colors in **Figure 3**, the detailed localization of each *Drosophila* PPP gene is summarized in **Figure S5**. The data collectively demonstrate that the PPP genes keep a well conserved position and orientation in the members of the melanogaster subgroup. On the other hand, many PPP genes, and first of all the novel type 1 phosphatase genes, frequently change positions in the more distantly related *Sophophora* and *Drosophila* subspecies. The details of three interesting rearrangements are summarized in **Figure S4**.

The *PpY-55A* gene evolved *via* an intron containing intermediate that was preserved in the members of the repleta, virilis, and willistoni groups (**Table 1**). The intron-containing *PpY-55A* disappeared in *D. grimshawi*, and lost its intron about 62 million years ago when the obscura and the melanogaster groups separated from the willistoni group (**Figure S4A**). In the obscura group the intronless version of *PpY-55A* relocated to element A (chromosome X) where it was subsequently duplicated (**Figure S5A**). The place of the translocated gene still can be recognized in element C (**Figure S4A**), but its sequence has been degraded. On the other hand, in the melanogaster group the new intronless *PpY-55A* gene replaced the old intron containing version in element C, but the direction of the coding strand got reversed due to a larger chromosomal inversion (**Figure S4B**).

In *D. pseudoobscura* and *D. persimilis* the *PpD5+* gene has a unique ∼60 bp intron in the coding region (**Tables 1**, **S1**). Neither the sequence, nor the position of this intron has been found in any PPP genes. This gene was deleted from the members of the melanogaster and willistoni groups, but its intronless version was established at the same location in all of the ancient *Drosophila* subspecies (**Table 1** and **Figure S4C, D**). Remarkably, the gain/loss of the intron was accompanied by gene inversion.

Synteny analysis revealed that in the melanogaster and the obscura groups *PpD5* has a well conserved localization in Muller element C between the landmark genes CG9308 and CG13500 (**Figure S4E**). The place of the gene can still be discerned in the other members of *Drosophilidae* but the gene sequence was degenerated (**Figure S4F**). However, *PpD5* was rescued in *D. willistoni* and *D. grimshawi*, as it was relocated to a new position in the same element between *cos* and *Eaf* in *D. mojavensis* and *D. virilis* (**Figure S4G**).

In agreement with the supplementary figure 6 of Koerich et al. [34] we found that several PPP genes, which are widely separated in *D. melanogaster*, form a cluster in the more ancient species (**Figure 5A**). We believe that the cluster of *D. virilis* represents the complete gene arrangement; 4 PPP genes are next to each other in Muller element C in the same orientation, following the order of *Pp1-Y1*, *PpD6+*, *PpD6*, and *Pp1-Y2*. The position of the genes suggests a series of tandem duplications that was probably initiated from *Pp1-Y2* that is closely related to the parental *Pp1-96A* (**Figure S2C, D**) and can be considered as the most ancient member of the cluster. In *D. grimshawi* the redundant *PpD6+* was eliminated, while in *D. mojavensis* both of the redundant genes *Pp1-Y1* and *PpD6+* were lost. In the willistoni and the obscura groups a central inversion reversed the first and second genes together, creating a new order of *PpD6+*, *Pp1-Y1*, *PpD6*, and *Pp1-Y2*. This gene cluster was mobilized and disintegrated in the melanogaster group. *PpD6+* was eliminated in all of the group members. In *D. ananassae* one half of the cluster i.e. *Pp1-Y2* and *PpD6* moved together into the Y chromosome [8], [34]. On the other hand, all of the cluster members were separated in the common ancestor of the melanogaster subgroup. *PpD6* landed in element B (**Figure 5A**) while *Pp1-Y1* and *Pp1-Y2* arrived to heterochromatic areas of Y [34].

### Adaptation of the PPP genes to the genomic environment in *Drosophilidae*

It has been reported previously that *Drosophila* genes residing in a heterochromatic environment have lower G−C content than their paralogs in euchromatic regions [35]. By extending the idea of gene adaptation we analyzed if the movements of the PPP genes between the autosomes and the heterochromatin rich Y affected their base composition. **Figure 5B** shows that the average G−C content of the PPP coding regions varies between 50–60%, except for *D. willistoni* that has a lower level of these nucleotides. The data follow the general tendency, and support the notion that *D. willistoni* has a lower G−C content because it prefers T over C in the codons of certain amino acids [36]. In accord with the expectation, the G−C content of the phosphatase cluster members is close to average when they are in Muller elements C or B, but the ratio becomes significantly reduced when they move to the Y (**Figure 5B**). By analyzing the G−C content of all known *Drosophila* genes that have been relocated between Y and other chromosomes we proved that the PPP genes obey a more general rule (**Table S3**).

## Discussion

### The functional rational behind the basic PPP toolkit of insects

When analyzing the origin of the large *Drosophila* PPP gene complement, it is inevitable to assume that a core set of indispensable PPP catalytic subunits must have been present in all of the insects. From the data of **Table 2** and **Figure 2** we concluded that the basic insect PPP set includes no more than 8 enzymes: 2 of the PPP1 isoforms and a single representative of each PPP that has a human ortholog numbered PPP2-7. The requirement for two type 1 phosphatases is not unexpected. It is known that animals posses at least two PPP1 isoforms, because one of them (the β/δ isoform) is specialized in muscular functions, while the other (the α isoform) operates in cell cycle regulation.

If we take into consideration that *Pp1-87B* and *Pp2B-14B* took over most of the roles of the more ancient *Pp1-97A* and *CanA1* phosphatases, respectively (see later), on the bases of molecular genetic studies carried out with *D. melanogaster* we can give the following functional explanation for the conservation of 8 core PPP enzymes: (i) The *flapwing (flw)* mutant exhibits aberrant flight muscle development [37] and an additional mitotic effect of *flw* has been recently suggested [38]. (ii) The inactivation of *Pp1-87B* causes a mitotic block in anaphase [38], [39]. The same gene is also involved in the interphase chromosome condensation [40] as well as in learning and memory [41]. (iii) The overexpression of active *Pp2B-14D*, results in lethality [42] and female sterility [43]. (iv) *Pp2A* is essential for the completion of the cell division cycle as witnessed by the *microtubule star* (*mts*) mutant in which the centromer and nuclear cycles are uncoupled [11], and chromosome segregation is impaired [38]. Furthermore, *mts* can mediate visual signaling [44], cytoskeletal organization and cell shape [45], phagocytosis [46], as well as the *sevenless* [47] and *hedgehog* [48] signal transduction pathways. (v) The role of *Pp4-19C* in microtubule organization was revealed by the analysis of the *centrosomes minus microtubules (cmm)* mutant [49]. In a systematic RNAi screen *Pp4-19C* was identified as a cell size regulator [38], and its involvement in neuroblast cell division has been proven [50], [51]. (vi) The accumulation of the *PpV* protein in embryos implicated this phosphatase in zygotic transcription and cellularization [13]. (vii) The modulator function of *PpD3* in mitotic cell cycle was suggested by Chen et al. [38]. (viii) *RdgC* acts in the G-protein mediated signaling pathway [52]. The accumulation of hyperphosphorylated rhodopsin in the *rdgC* mutant induces the degeneration of the photoreceptor cells by apoptosis [53], [54]. Obviously, the members of the core set of PPP enzymes acquired important if not essential functions and got fixed in most of the insect genomes (**Table 2**) including all of the Drosophila species (**Table 1**). These 8 genes represented the starting material for retropositions and subsequent tandem duplications. Based on the available data including DNA and protein sequence analysis, chromosomal localization, and expression patterns of the genes we put together a hypothetical sequence of events that expanded the PPP enzyme family in *Drosophilidae*.

### The duplication of classical PPP enzymes produced the first generation of functional PPP retrogenes

The basic PPP toolkit was expanded by the duplication of the classical calcineurin and type 1 phosphatases (**Table 1** and **Figure 3**) using at least in part an unorthodox, RNA intermediate based retroposition mechanism [reviewed in 55]. The first retroduplication took place in *Diptera* and produced *Pp2B-14D* that was later duplicated again in *Drosophilidae* (**Figure 4**). Betran et al. [56] reported that in *D. melanogaster CanA-14F* originated from *CanA1 via* retroposition. Later it was reported that *Pp2B-14D* was also a retrogene that was derived from *CanA1* [57]. A careful inspection of the database (http://flybase.org/) revealed that -in contrast with the original designation- *Pp2B-14D* falls into the 14E1-14E3 chromosomal subdivision. Since *CanA-14F* is at 14E3-14F1 they are next-door neighbors in chromosome X (**Figure 1B**). The revised chromosomal localization suggests that one of these genes was generated from the other by local gene duplication. As *Pp2B-14D* appeared first in *Diptera*, while *CanA-14F* is found only in *Drosophilidae* (**Figure 4**) it is logical to assume that the tandem duplication of the older *Pp2B-14D* gene resulted in the more recent *CanA-14F* copy. This sequence of events is supported by the fact that in *D. melanogaster Pp2B-14D* is the most predominant calcineurin isoform [10] that has important functions [42], [43]. On the other hand, a P-element insertion mutant of *CanA-14F* has no obvious phenotypes (http://flybase.org/). Curiously, *Drosophila* also kept the ancestral intron containing *CanA1* gene, probably because its gene product gained a new function in the innate immune response [58]. In contrast, in other members of *Diptera* the predominant *Pp2B-14D* retrogene took over the important calcineurin functions, and eventually replaced the parental gene.

The second retroposition based duplication event took place in the ancestor of all *Drosophila* lineages and produced *Pp1-87B* from *Pp1-96A* [57]. Due to the very strong sequence similarity it is more difficult to decide if *Pp1-13C* originated directly from *Pp1-96A*, or indirectly via *Pp1-87B* (**Figure S2D**). In *D. melanogaster Pp1-87B* is an essential, predominant PPP1 isoenzyme form [59], while the inactivation of *Pp1-96A* does not result in any obvious phenotype [60]. However, the properties of the *Pp1-96A/flw* double mutant indicate a functional overlap between the two paralogs. As the overexpression of *Pp1-13C* did not affect either *Pp1-87B* or *flw* mutants it seems to be a redundant gene product [60].

### A second wave of retropositionos and tandem duplications created novel *Drosophila* specific PPP retrogenes

According to our reconstruction *Pp1-96A* was the forefather of a large family of novel type 1 phosphatase retrogenes (**Table 1** and **Figure 3**). The tracing of the evolutionary history of this large subfamily proved to be difficult, since the traditional phylogenetic analysis repeatedly resulted in uncertain branching points in the family trees (**Figures 2** **,** **3**). The reduction of the dataset to the type 1 PPP proteins did not help resolving the problem (not documented results), that is why these sequences were also analyzed by a multidimensional sequence scaling method (**Figure S2C, D**). In the two dimensional representation of this comparisons the distance between two dots (representing two individual proteins) is proportional to the sequence similarity. According to gene structures *Pp1-96A* can be regarded as the parent of the partially retroposed *PpY-55A* gene that retained one of its introns (see later). As discussed before, the duplication of *Pp1-96A* created *Pp1-87B*, the first perfectly processed retrogene in the subfamily. Either *Pp1-96A* or *Pp1-87B* could have been the starting points of additional retropositions that resulted in the classical *Pp1-13C*, as well as in the novel *Pp1-Y2* and *PpD5* phosphatases (**Figure S2C, D**). Multidimensional sequence scaling data suggest that *PpD5+* was generated from *PpD5* meanwhile the duplication of *Pp1-Y2* produced both *PpD6* and *PpN58A*. In turn *PpD6* duplicated to give rise to *Pp1-Y1* and *PpD6+* (**Figure S2C**). The close relationship between *Pp1-Y2*, *PpD6*, *Pp1-Y1*, and *PpD6+* genes is supported by their juxtaposed chromosomal localization (**Figure 5A**). The exact timing of these 11 duplication events is not known, but they must have happened in the common ancestor of the *Drosophilidae* more that 63 million years ago (**Figure 4**).

Additional retroposition events extended the PPP family in a subspecies or group of *Drosophilidae*. 44–63 million years ago a new *Pp6+* variant was generated from the ancient ortholog of *PpV/Pp6* in the *Drosophila* subspecies. This novel retrogene was lost in the *Sophophora* members. A more specific set of PPP duplications was restricted to the obscura group. Around 62 million years ago the retroposition of *Pp4* generated the obscura group specific *Pp4+*. At the same time the repositioning and duplication of *PpY-55A* gene resulted in the new *PpY+*. Meanwhile the intron-containing parental gene disappeared and was replaced by an intronless *PpY-55A*. Finally, 44–55 million years ago the last retroposition in the melanogaster group produced *CG11597* from *Pp4*. It is also clear, that *Pp4+* is distinct from *CG11597* and can not be considered as its precursor. Thus, the ancestral *Pp4-19C* gene underwent two separate retropositions in the melanogaster and in the obscura groups, resulting in *CG11597* and *Pp4+*, respectively (**Figure 4**).

### The novel functional PPP retrogenes exhibit male biased expression

We have reported that the male biased transcription of the type 1 *PpY-1*, *PpY-2*, *PpD5*, *PpD6*, *PpN58A* and *PpY-55A* retrogenes was conserved during the evolution of *Drosophilidae* [8]. Here we extend this observation and prove that 5 more of the novel functional PPP retrogenes: *PpD5+*, *PpD6+*, *PpY+*, *Pp4+*, and *Pp6+* have the same male specific expression pattern (**Table 3** and **Figure S3**). Our results are in good agreement with the observation that many of the *Drosophila* retrogenes are preferentially expressed in the male germline [55], [57], and the new male biased PPPs evolve faster [8]. 4 out of the 5 new male-biased PPP genes are located on autosomes, only *PpY+* resides in Muller element A that corresponds to the left arm of chromosome X in *D. pseudoobscura* (**Table 1**). Furthermore, the parental *Pp4-19C* and *PpV*, *Pp6* genes are in Muller element A (chromosome X) in all of the 12 *Drosophila* species. Consequently, their retrogene copies moved out of X support the thesis that male-specific retrogenes tend to avoid X inactivation throughout the *Drosophila* lineages [55], [56], [61].

Our earlier publication [8] and present data (**Table 3**) together suggest that most of the recent PPP functional retrogenes follow the “out of testis” hypothesis of Vinckenbosch et al. [62]. While the older (classical or first generation) PPP retrogenes developed a differential expression pattern and gained specialized functions, 11 younger (novel or second generation) PPP retrogenes are still male specific. As usual, there is one exception to the rule, one of the youngest PPP members, *CG11597* is unbiased, it is transcribed in different developmental stages and in both sexes [8].

Save for their male specific expression, we have no information on the functions of the 5 new PPP members. As a matter of fact the same holds for the 6 other novel PPP retrogenes, whose transcription in the testis of *D. melanogaster* was demonstrated earlier [8]. Most probably they are in the early stages of their evolution; and they perform overlapping or redundant roles. We propose, that a sizable pool (6–10 genes) of still actively changing novel PPP retrogenes provides a powerful reservoir for the evolution of new regulatory mechanisms. The faster evolution of the novel PPP retrogenes is demonstrated by their higher rate of nonsynonymous nucleotide substitutions in comparison to the older classical PPP genes (**Table S4**). Most of the latter are expressed in the testis of the males that is a shelter and a testing ground of the fast evolving novel genes [62]. According to this hypothesis the advantageous gene variants can be selected through the improved reproductive fitness of the males.

### The novel PPP retrogenes rearrange and move dynamically in *Drosophila* genomes

While investigating the rearrangements of the PPP genes we found examples for various unorthodox retroposition events [57] like: partial retroposition, chimeric retrogene formation, retroposition followed by relocation, and retroposition followed by tandem duplications. The great variety of the molecular genetic arsenal utilized underlines a vigorous struggle of the novel PPP retrogenes to survive in the tough competition with their parental and sister phosphatases (**Figures 5**, **S3**). We found the following thee interesting examples for gene rearrangements/translocations.

- In the ancient form of *PpY-55A* one short intron of the parental *Pp1-96A* was retained in a well conserved position of the new retrogene due to the reverse transcription of a partially processed mRNA. Similar partial retropositions of 6 other genes were reported before by Bai et al. [57]. The intron was lost in the obscura and melanogaster groups *via* two different mechanisms. In *D. pseudoobscura* and *D. persimilis* retroposition placed the intronless *PpY-55A* into Muller element A, where it was subsequently duplicated giving rise to the intronless *PpY+*. Meanwhile the intron containing gene copy got degraded. Obviously, the intronless genes in element A took over the functions of the older gene. In the melanogaster group the intron was lost *via* the recombination of a fully processed cDNA with the short intron containing parental gene [63].
- The evolutionary history of *PpD5+* is even more complicated. This gene encompasses a unique short intron in the members of the obscura group. It is possible, that this new intron was picked up from the flanking region of the insertion site, thus according to the definition of Bai et al. [57] *PpD5+* may represent one of the few chimeric retrogenes. Later on, in all of the ancient *Drosophila* subspecies an intronless retrogene replaced the intron containing copy at the same location. Alternatively, the intronless copy of *PpD5+* was generated first, and gained a small intron in the obscura group [63]. Disregarding the mechanism of the intron loss or gain the *PpD5+* gene was deleted from the members of the melanogaster and willistoni groups (**Table 1**).
- The movements of *PpD5* as well as the rearrangements and final disintegration of the ancient *Pp1-Y1*, *PpD6+*, *PpD6*, *Pp1-Y2* gene cluster exemplify the relocation of complete PPP genes without the modifications of the gene structures. One important aspect of the gene movements is the translocation of PPP genes into the heterochromatic Y chromosome [34]. It is interesting to see that 3 out of the 4 PPP gene cluster members acquired transient or permanent localization in Y. We noted that the jumping of these retrogenes into the heterochromatic environment was accompanied wit the decrease of G−C content in the ORFs. The changes in the base composition of *PpD6* are especially instructive, as this gene moved from element C to Y in *D. ananassae* and than to B element in the melanogaster subgroup [8]. In agreement with Diaz-Castillo and Golic [35] our results show that the PPP genes adapted their G−C content to the changing genomic environment. The modification of codon usage, and first of all the changes in third codon positions reduces the G−C content of the coding regions and allows the expression of the phosphatase genes even from the heterochromatic chromosome Y.

In conclusion, the relatively small PPP gene family, like a drop of the sea, reflects many colorful molecular events of evolution. Our work demonstrates that retropositions, tandem duplications, deletions and relocations have steadily modified the PPP repertoire of the fruit flies. From this respect *Drosophilidae* is an especially resourceful organism as it accumulated the largest PPP complement in the animal kingdom. The dynamic alterations including the changes of the numbers, structures, orientation, and chromosomal localization of PPP genes contributed to the genetic diversity in *Drosophilidae*.

## Supporting Information

Figure S1 The protein identifications for 287 insect PPP catalytic subunits and the bootstrap values corresponding to **Figure 2** are shown. The color codes of the proteins from non-*Drosophila* species are the same as in **Figure 2**. Uncertain branching points are circled.
(PDF)

Figure S2 **Phylogeny of 12** ***Drosophila*** **species and their PPP catalytic subunits.** (**A**) The family tree of 12 *Drosophila* species was constructed on the bases of the mutational clock determined by Tamura et al. [64] and Koerich et al. [34]. The main subspecies, groups and subgroups are labeled. (**B**) The protein identifications of 227 *Drosophila* PPP catalytic subunits and the bootstrap values corresponding to **Figure 3** are shown. The branches of the tree are colored according to the chromosomal localization of the appropriate gene. The color codes of Muller elements are given in the lower right corner for reference. Uncertain branching points are circled. All of the protein names and sequences are given in **Table S2**. (**C**) Comparison of the amino acid sequences of 126 type 1 protein phosphatase catalytic subunits by a multidimensional scaling method. In the scatter-plot each point represents one PPP. Orthologs are circled, except for the *PpY-55A*, *PpY+* circle that contains 2 paralogs. 7 PPP sequences (Dana *PpD6_R*, Dper *flw*_R, Dper *Pp1-Y1*_R Dsec *Pp1-Y1*_S, Dsec *Pp1-Y2*_R, and Dsim *Pp1-Y2*_R) fall outside of the +/−2.5 range, and are not depicted in the figure. (**D**) The box in (C) is exploded. Orthologs are circled but *Pp1-96A* and *Pp1-87B* sequences are intermixed in one circle.
(PDF)

Figure S3 The sex specific expression of PPP genes in *D. melanogaster* (**A**), *D. ananassae* (**B**), *D. pseudoobscura* (**C**), *D. willistoni* (**D**), and *D. virilis* (**E**) imagos was determined by RT-PCR. *RpL23* was used as an internal control. Genomic DNA (G) was the target in the control PCR. + denotes RT-PCR, and − stands for PCR alone (negative control, without RT reaction) with the appropriate RNA preparations. S labels a 100 bp DNA ladder in which the strongest 500 bp band is marked. S2 labels a 1 kbp DNA ladder.
(PDF)

Figure S4 **Analysis of specific PPP gene movements in** ***Drosophilidae*** **.** The synteny of *PpY-55A*, *PpD5+*, and *PpD5* genes are shown in panels (**A**), (**C**), and (**E**). Homologous chromosomal regions of about 200 kbp are represented by double headed arrows. Abridged species names are on the left, chromosome/scaffold identifications and ranges are either on the right side or in the middle of the panels. Broken lines indicate large DNA segments that are situated between the two depicted areas. Arrows show the direction and size of landmark genes, **o** labels the expected position of a missing gene. The names of the intronless genes are boxed. Dot plots compare homologous chromosomal regions containing the *PpY-55A* (**B**), *PpD5+* (**D**), and *PpD5* (**F, G**) genes from selected *Drosophila* species. In (**B**) broken lines delimit two large inversions, which are circled in the plot. The inverted *PpY-55A* gene is boxed. In (**D**) a small arrow at the right side of the plots shows the size and direction of the *PpD5+* gene in *D. virilis*. This gene is expected to occur in the areas between the two horizontal lines. The inverted *PpD5+* gene is boxed in *D. pseudoobscura*. The corresponding gene region was deleted from *D. ananassae* and *D. willistoni*. In (**F**) the arrow shows the size and direction of the *PpD5* gene in *D. persimilis*. This gene is expected to occur in the areas between the two horizontal lines, but can not be recognized in *D. grimshawi* and *D. willistoni* because its sequence has been degraded. Panel (**G**) demonstrates that the chromosomal region in question is missing from *D. grimshawi* and *D. willistoni* indicating that *PpD5* was inserted into this location in *D. mojavensis*. The scale indicates 10 kbp in all panels.
(PDF)

Figure S5 Chromosomal localization of PPP genes in Muller elements A (**A**), B (**B**), C (**C**), D (**D**), and E (**E**) of 12 *Drosophila* species. Abridged species names are given at the right side of the panels. Horizontal open bars represent continuous chromosomes or chromosome arms and a gap indicates a missing DNA sequence. A vertical line shows the localization, and the arrow tell the direction (left to right is 5′ to 3′ in the upper strand) of a given gene. The scale bar is 2 Mbp in all cases.
(PPT)

Table S1 The sequences of oligonucleotides and the experimental conditions used for PCR or RT-PCR are summarized in three sections. (**A**) Oligonucleotide primers and conditions used for the detection of *Drosophila* PPP transcripts by RT-PCR. (**B**) Oligonucleotide primers and conditions used for the amplification and sequencing of *Drosophila* PPP genes. (**C**) Oligonucleotide primers and conditions used for the amplification of *Drosophila RpL23* in control experiments.
(DOC)

Table S2 Predicted amino acid sequences of PhosphoProtein Phosphatase catalytic subunits in 12 *Drosophila* species are organized in separate Excell worksheets according to the enzyme names. The suffix S after a protein name means that the amino acid sequence was predicted from a DNA sequence determined in the present study. The suffix R indicates that the sequence was revised as explained in the linked attachment. Hyperlinks and Notes contain additional information on the polypeptides. Localization tells the Muller element that encompasses the corresponding gene. Protein sequences are in FASTA format, X stands for an unidentified amino acid residue.
(XLS)

Table S3 The G−C content in % is given for the coding regions of all *Drosophila* genes that changed location between Y chromosome and other (somatic or X) chromosomes.
(DOC)

Table S4 The evolution of PPP genes in *Drosophila* was analyzed by comparing the dn/ds values of the classical and novel PPP enzymes.
(XLS)
