# Supplementary material for: Molecular Evolution of Phosphoprotein Phosphatases in Drosophila
Source: PLoS One. 2011 Jul 15;6(7):e22218. doi: 10.1371/journal.pone.0022218 (PMC3137614; doi:10.1371/journal.pone.0022218)

Supplementary Fig. S2A

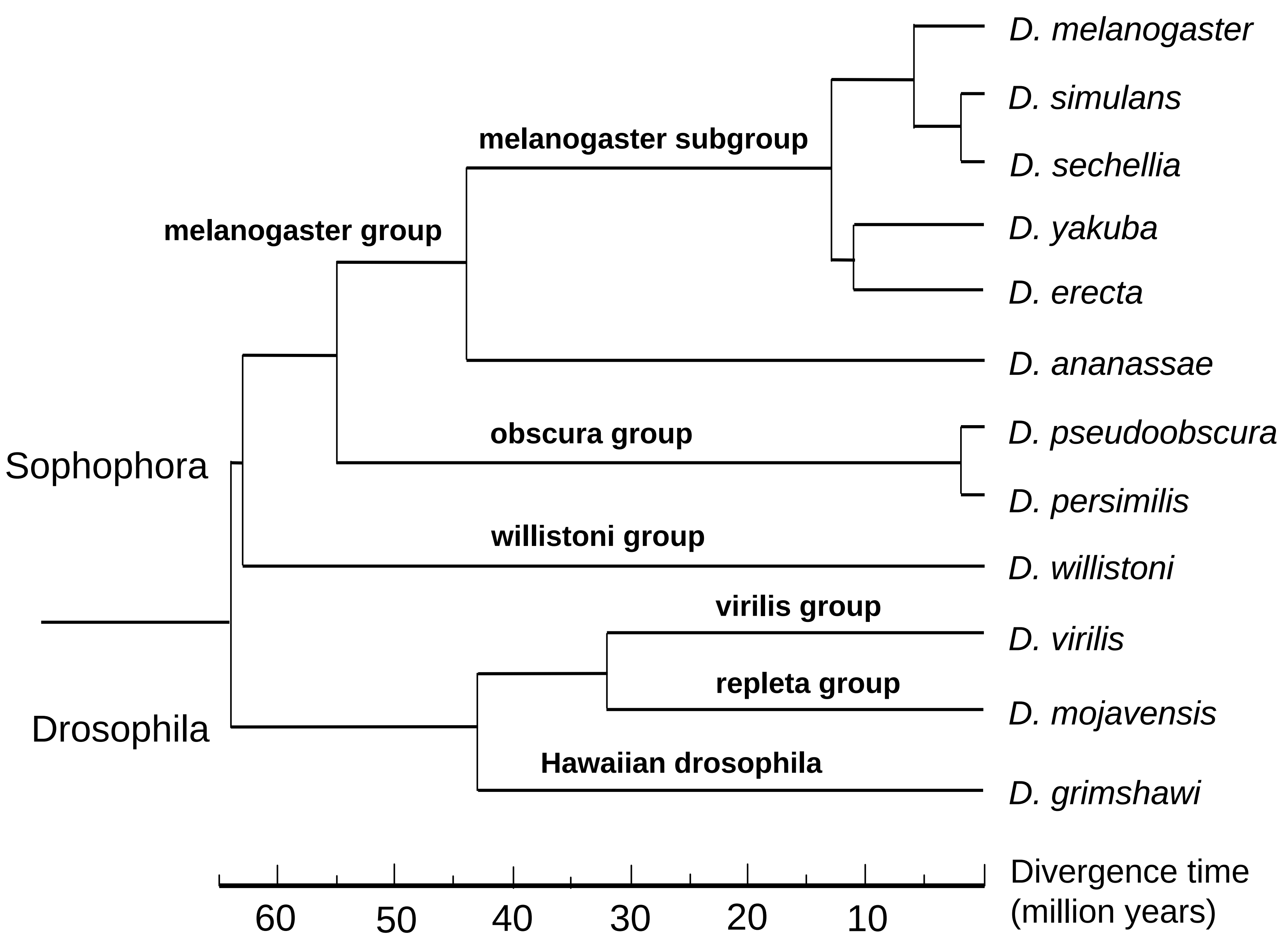

Supplementary Fig. S2B

Drosophila PPPs

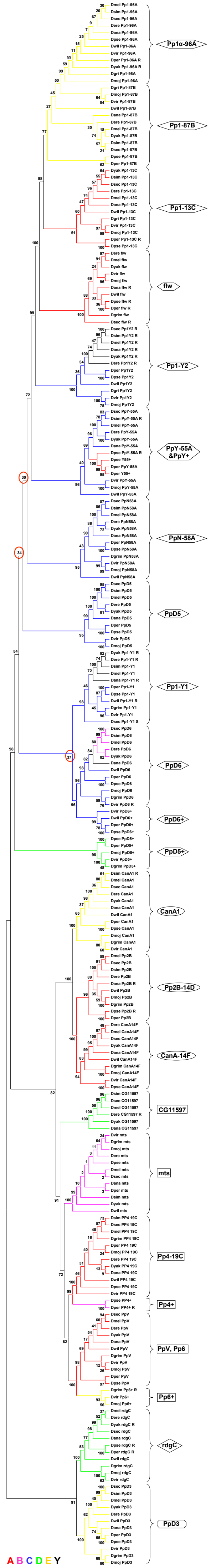

A B C D E Y

Supplementary Fig. S2C

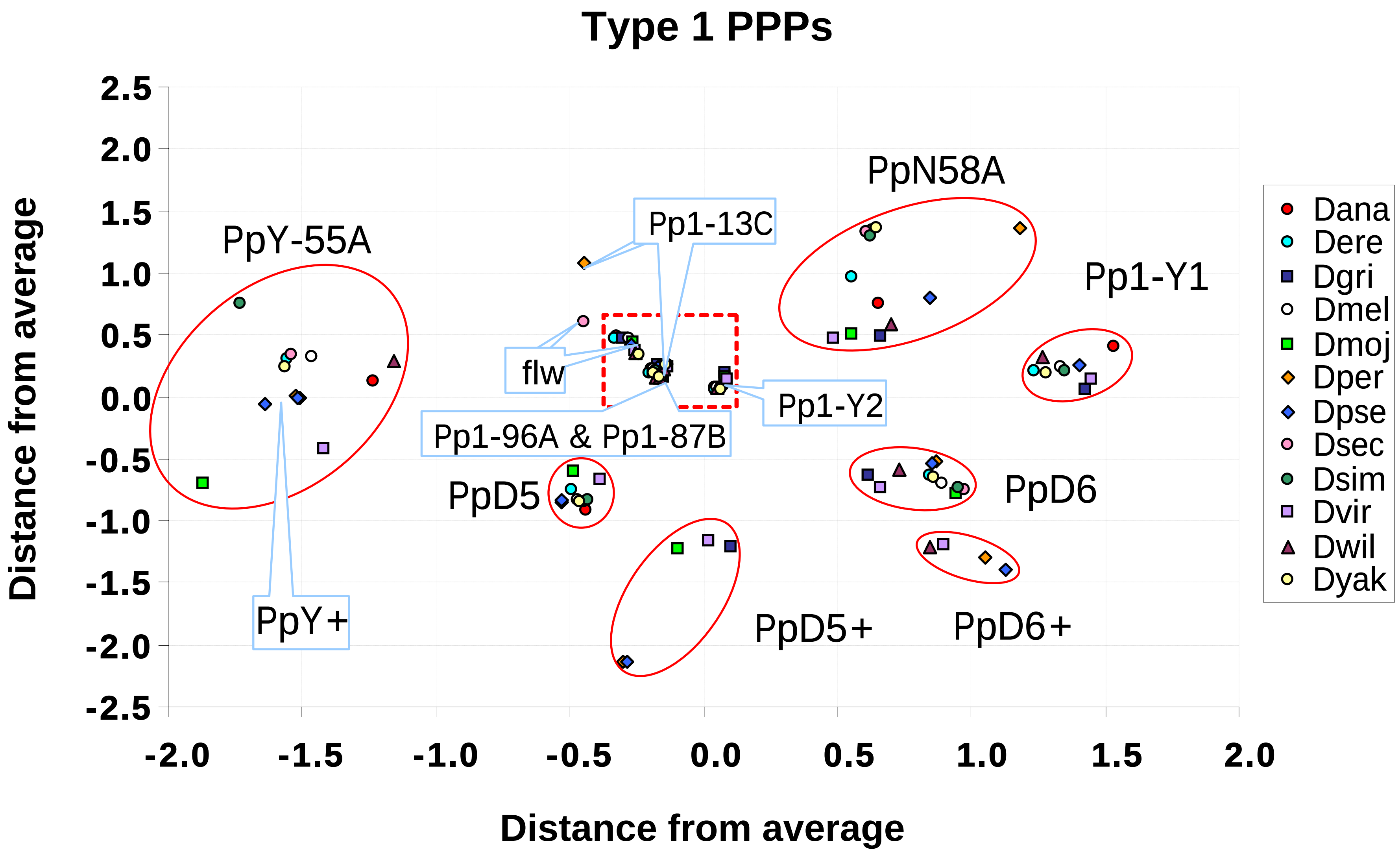

Supplementary Fig. S2D

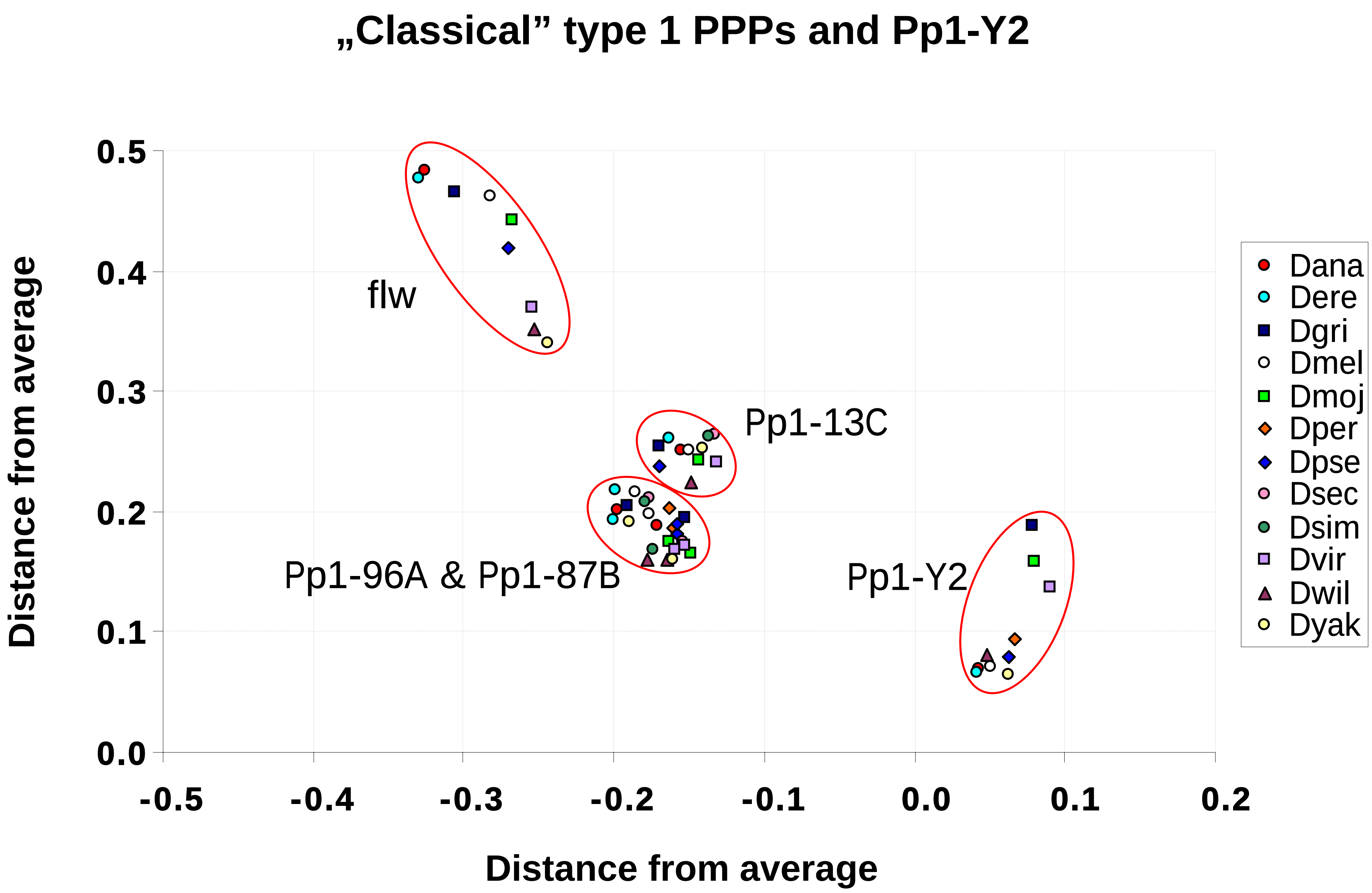

Supplement: Figure S2 — Phylogeny of 12 Drosophila species and their PPP catalytic subunits. (A) The family tree of 12 Drosophila species was constructed on the bases of the mutational clock determined by Tamura et al. [64] and Koerich et al. [34]. The main subspecies, groups and subgroups are labeled. (B) The protein identifications of 227 Drosophila PPP catalytic subunits and the bootstrap values corresponding to Figure 3 are shown. The branches of the tree are colored according to the chromosomal localization of the appropriate gene. The color codes of Muller elements are given in the lower right corner for reference. Uncertain branching points are circled. All of the protein names and sequences are given in Table S2. (C) Comparison of the amino acid sequences of 126 type 1 protein phosphatase catalytic subunits by a multidimensional scaling method. In the scatter-plot each point represents one PPP. Orthologs are circled, except for the PpY-55A, PpY+ circle that contains 2 paralogs. 7 PPP sequences (Dana PpD6_R, Dper flw_R, Dper Pp1-Y1_R Dsec Pp1-Y1_S, Dsec Pp1-Y2_R, and Dsim Pp1-Y2_R) fall outside of the +/−2.5 range, and are not depicted in the figure. (D) The box in (C) is exploded. Orthologs are circled but Pp1-96A and Pp1-87B sequences are intermixed in one circle. (PDF) [file pone.0022218.s002.pdf]
