# Supplementary material for: Molecular Evolution of Phosphoprotein Phosphatases in Drosophila
Source: PLoS One. 2011 Jul 15;6(7):e22218. doi: 10.1371/journal.pone.0022218 (PMC3137614; doi:10.1371/journal.pone.0022218)

Figure S3A

Drosophila melanogaster

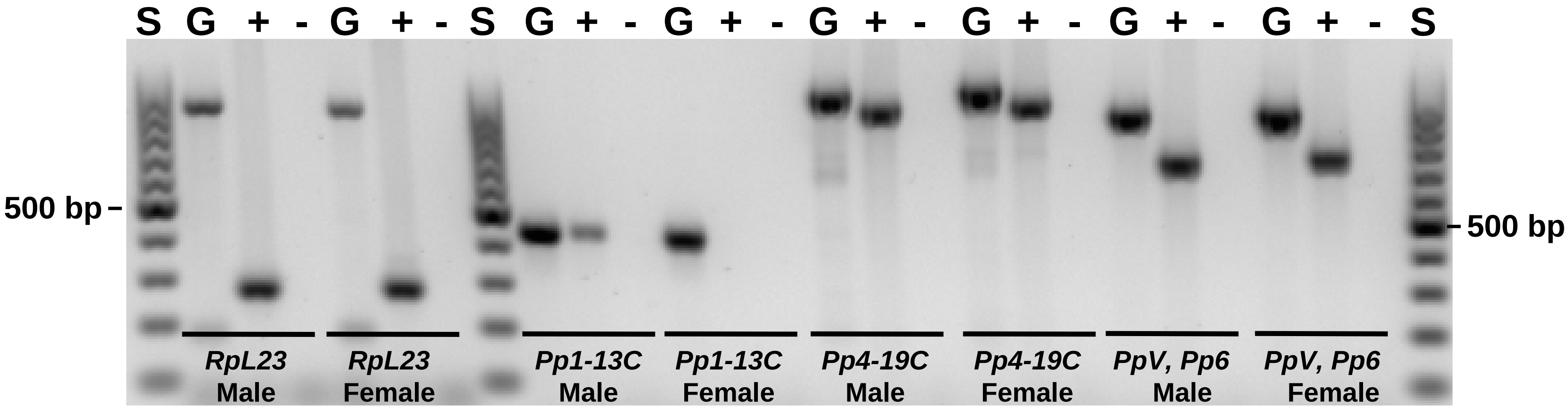

Figure S3B

Drosophila ananassae

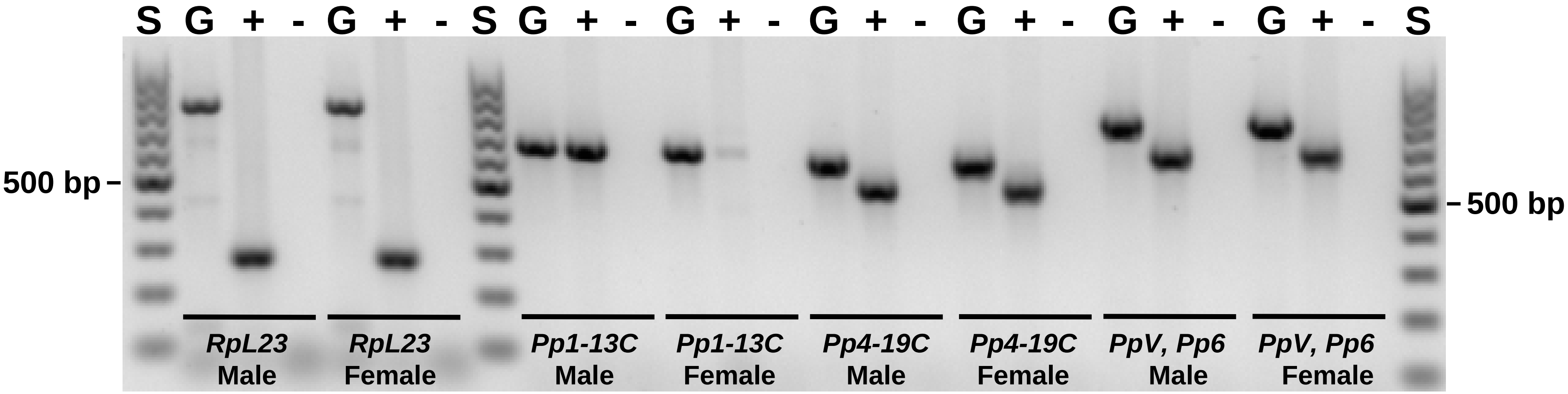

Figure S3C

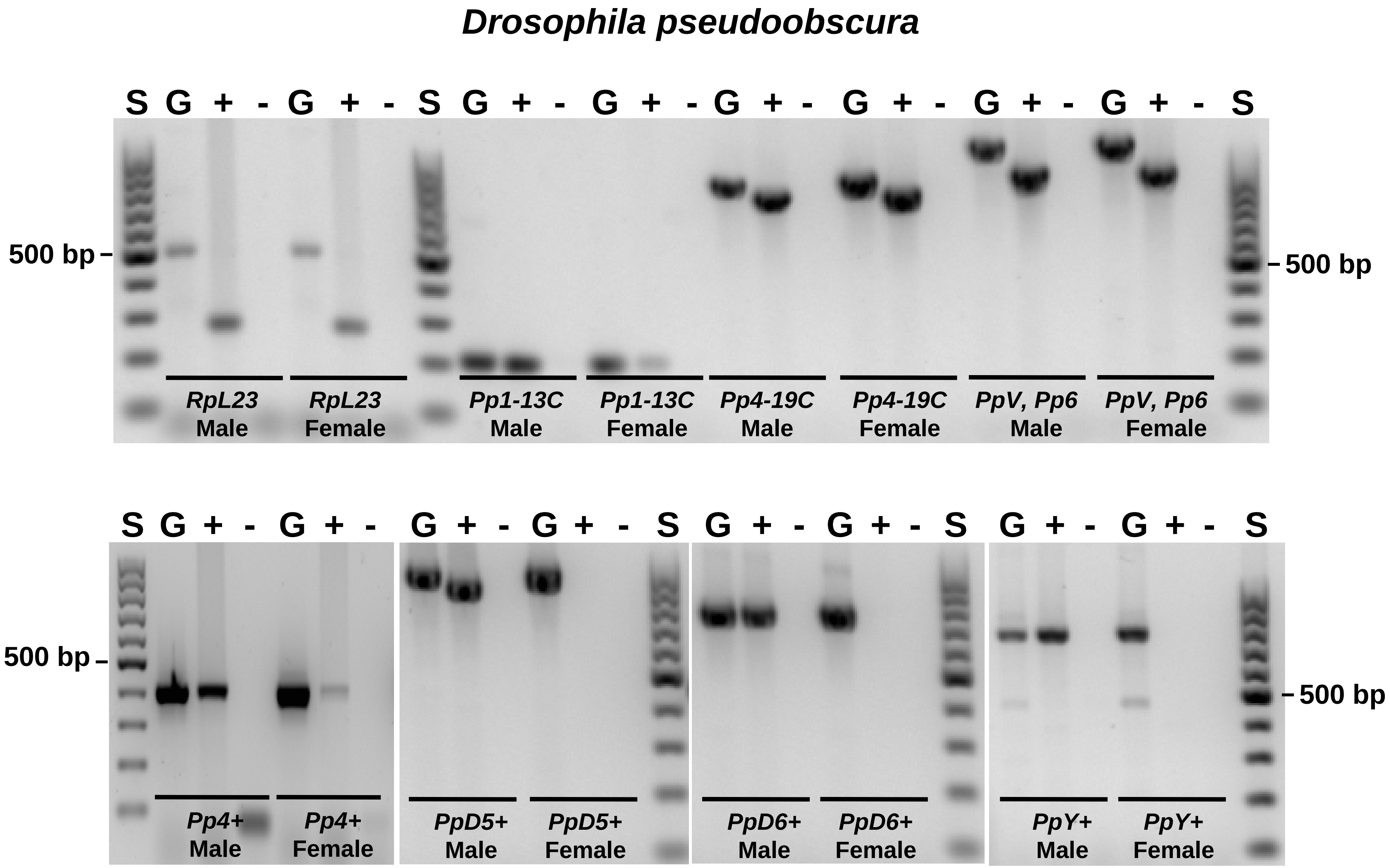

Drosophila pseudoobscura
S G + - G + - S G + - G + - G + - G + - G + - G + - S
500 bp
500 bp
RpL23 Male
RpL23 Female
Pp1-13C Male
Pp1-13C Female
Pp4-19C Male
Pp4-19C Female
PpV, Pp6 Male
PpV, Pp6 Female
S G + - G + - G + - G + - S G + - G + - S G + - G + - S
500 bp
500 bp
Pp4+ Male
Pp4+ Female
PpD5+ Male
PpD5+ Female
PpD6+ Male
PpD6+ Female
PpY+ Male
PpY+ Female

Figure S3D

Drosophila willistoni

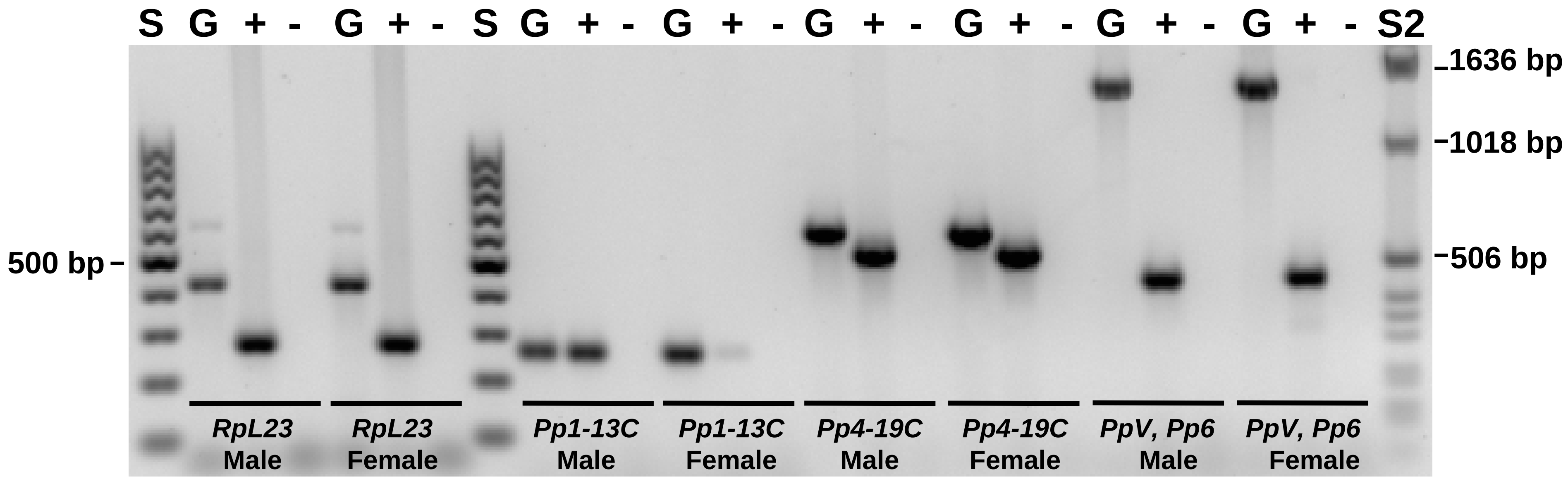

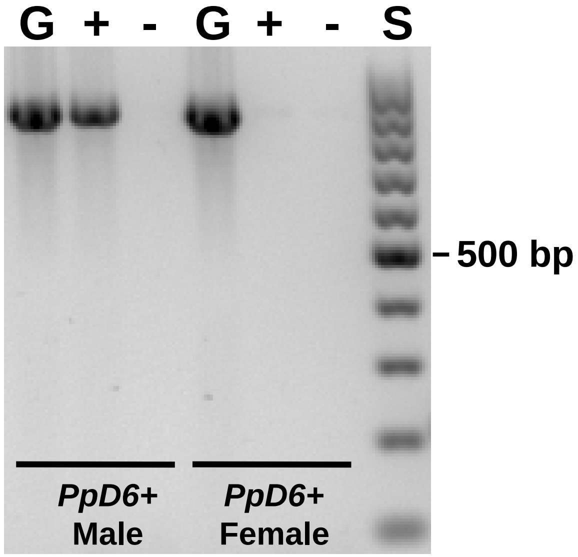

Figure S3E

Drosophila virilis

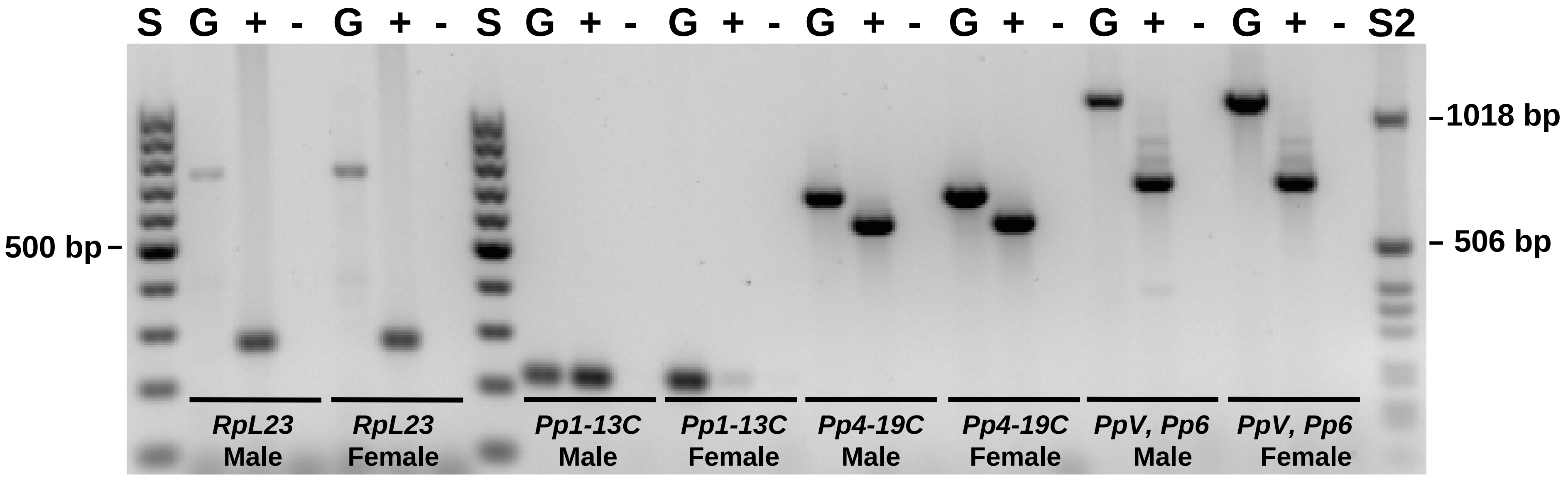

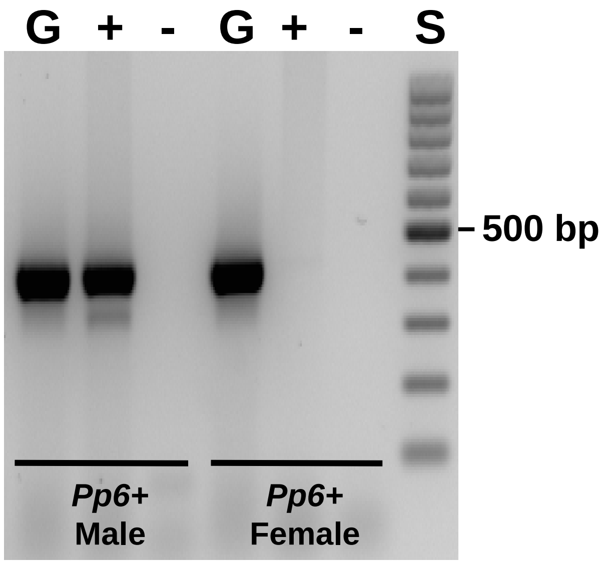

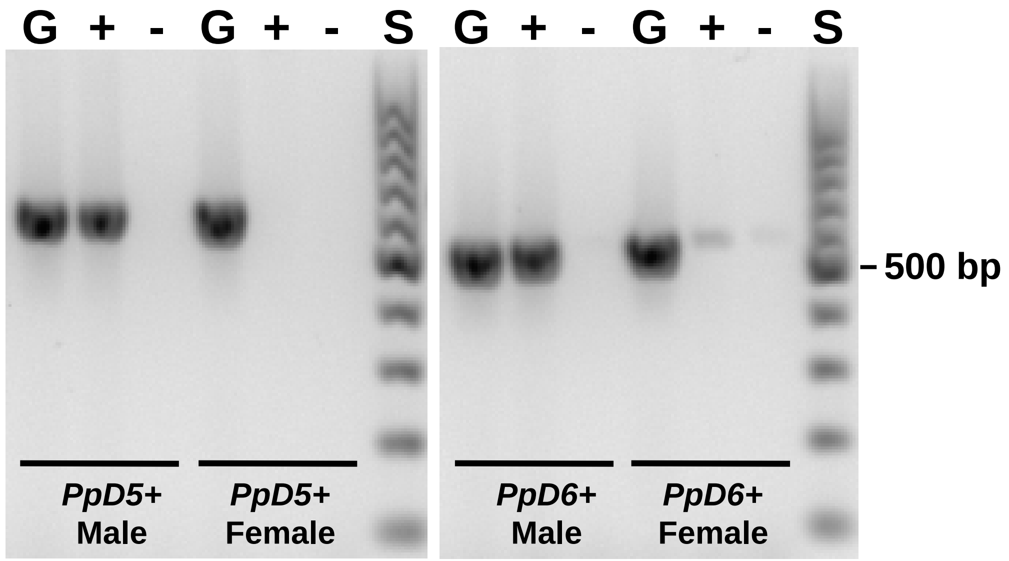

Supplement: Figure S3 — The sex specific expression of PPP genes in D. melanogaster (A), D. ananassae (B), D. pseudoobscura (C), D. willistoni (D), and D. virilis (E) imagos was determined by RT-PCR. RpL23 was used as an internal control. Genomic DNA (G) was the target in the control PCR. + denotes RT-PCR, and − stands for PCR alone (negative control, without RT reaction) with the appropriate RNA preparations. S labels a 100 bp DNA ladder in which the strongest 500 bp band is marked. S2 labels a 1 kbp DNA ladder. (PDF) [file pone.0022218.s003.pdf]
