# Supplementary material for: Molecular Evolution of Phosphoprotein Phosphatases in Drosophila
Source: PLoS One. 2011 Jul 15;6(7):e22218. doi: 10.1371/journal.pone.0022218 (PMC3137614; doi:10.1371/journal.pone.0022218)

Figure S4A

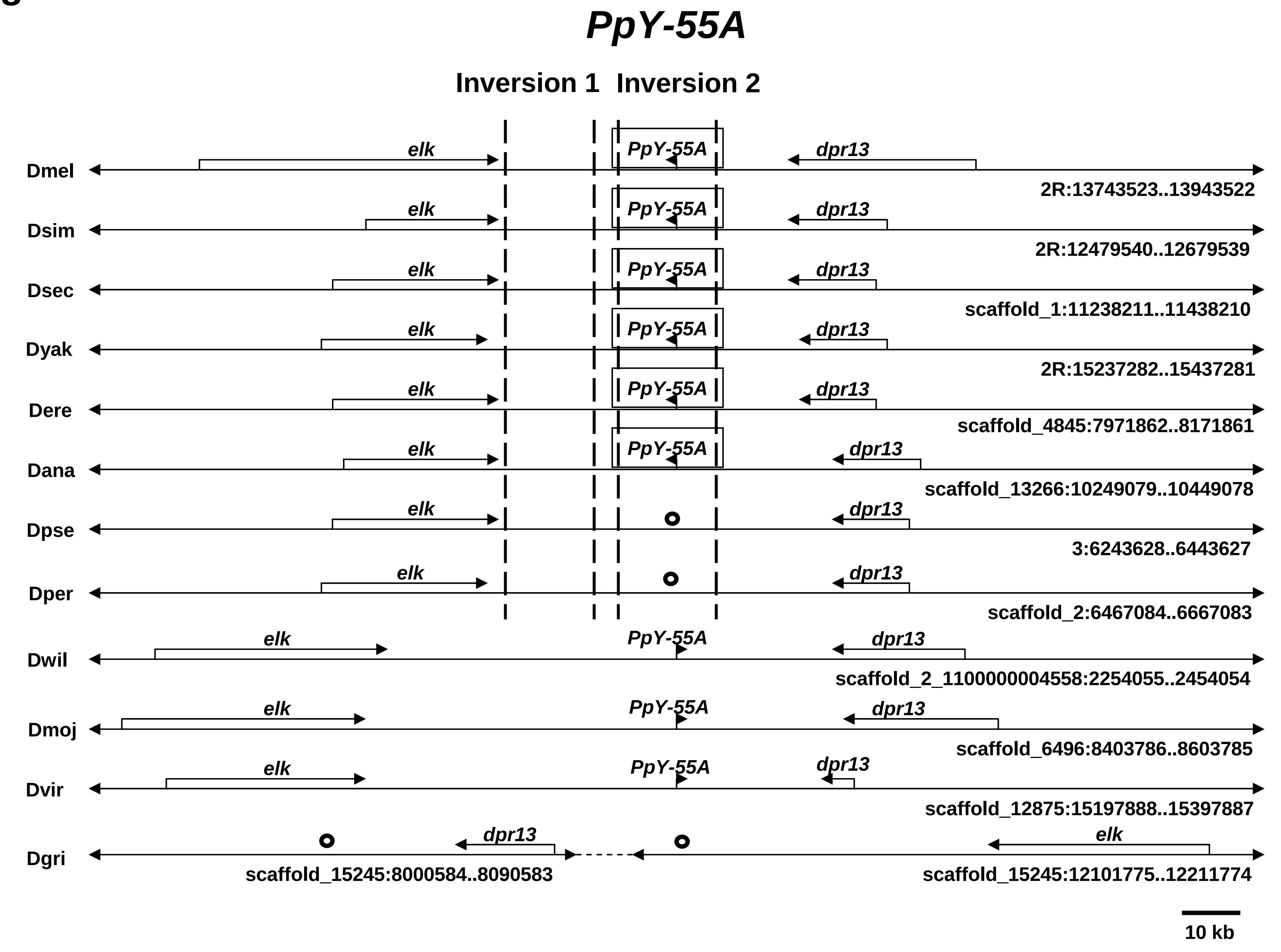
PpY-55A
Inversion 1
Inversion 2
Dmel
elk
PpY-55A
dpr13
2R:13743523..13943522
Dsim
2R:12479540..12679539
Dsec
scaffold_1:11238211..11438210
Dyak
2R:15237282..15437281
Dere
scaffold_4845:7971862..8171861
Dana
scaffold_13266:10249079..10449078
Dpse
3:6243628..6443627
Dper
scaffold_2:6467084..6667083
Dwil
scaffold_2_1100000004558:2254055..2454054
Dmoj
scaffold_6496:8403786..8603785
Dvir
scaffold_12875:15197888..15397887
Dgri
scaffold_15245:8000584..8090583
scaffold_15245:12101775..12211774
10 kb

Figure S4B

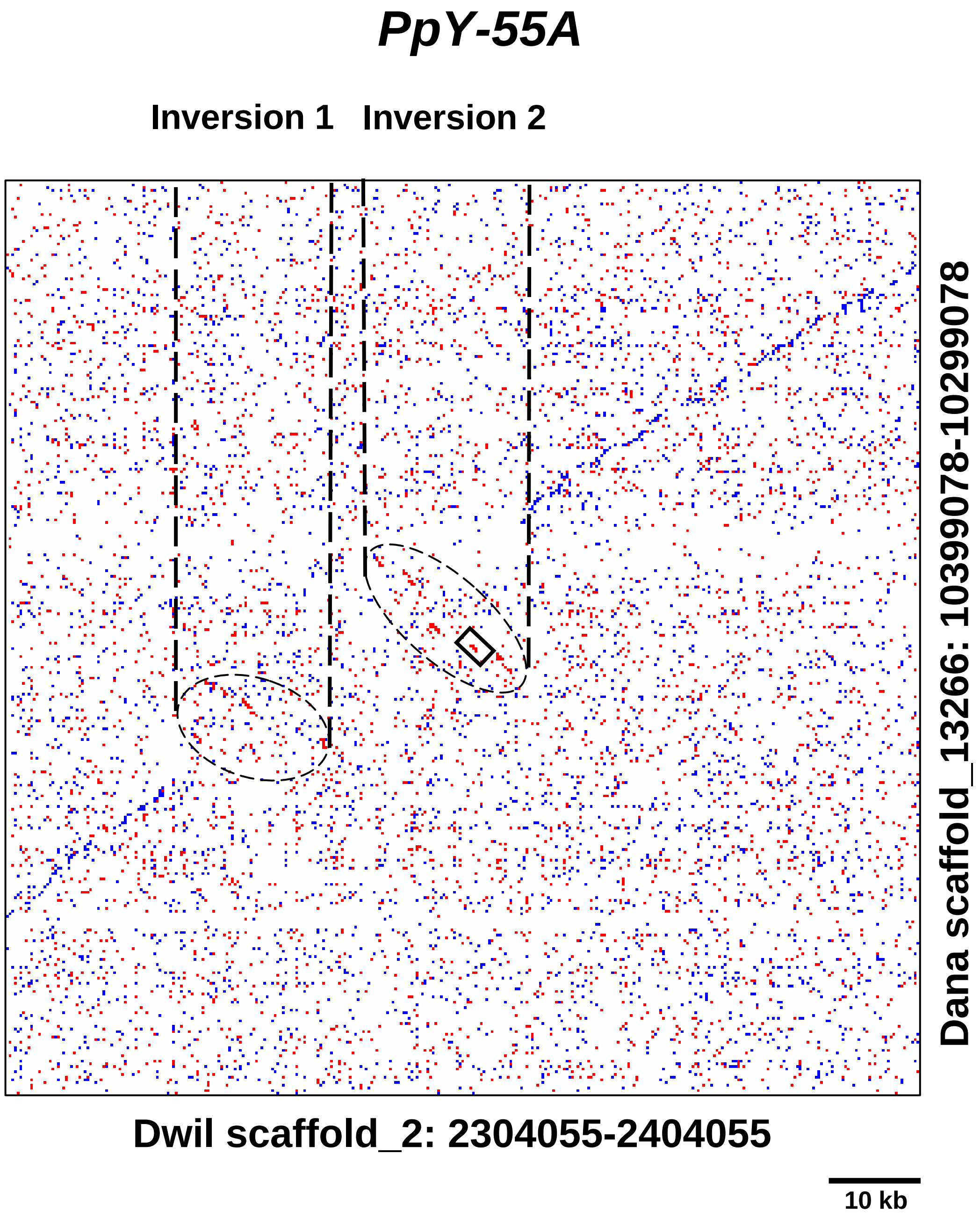

PpY-55A
Inversion 1
Inversion 2
Dana scaffold_13266: 10399078-10299078
Dwil scaffold_2: 2304055-2404055
10 kb

Figure S4C

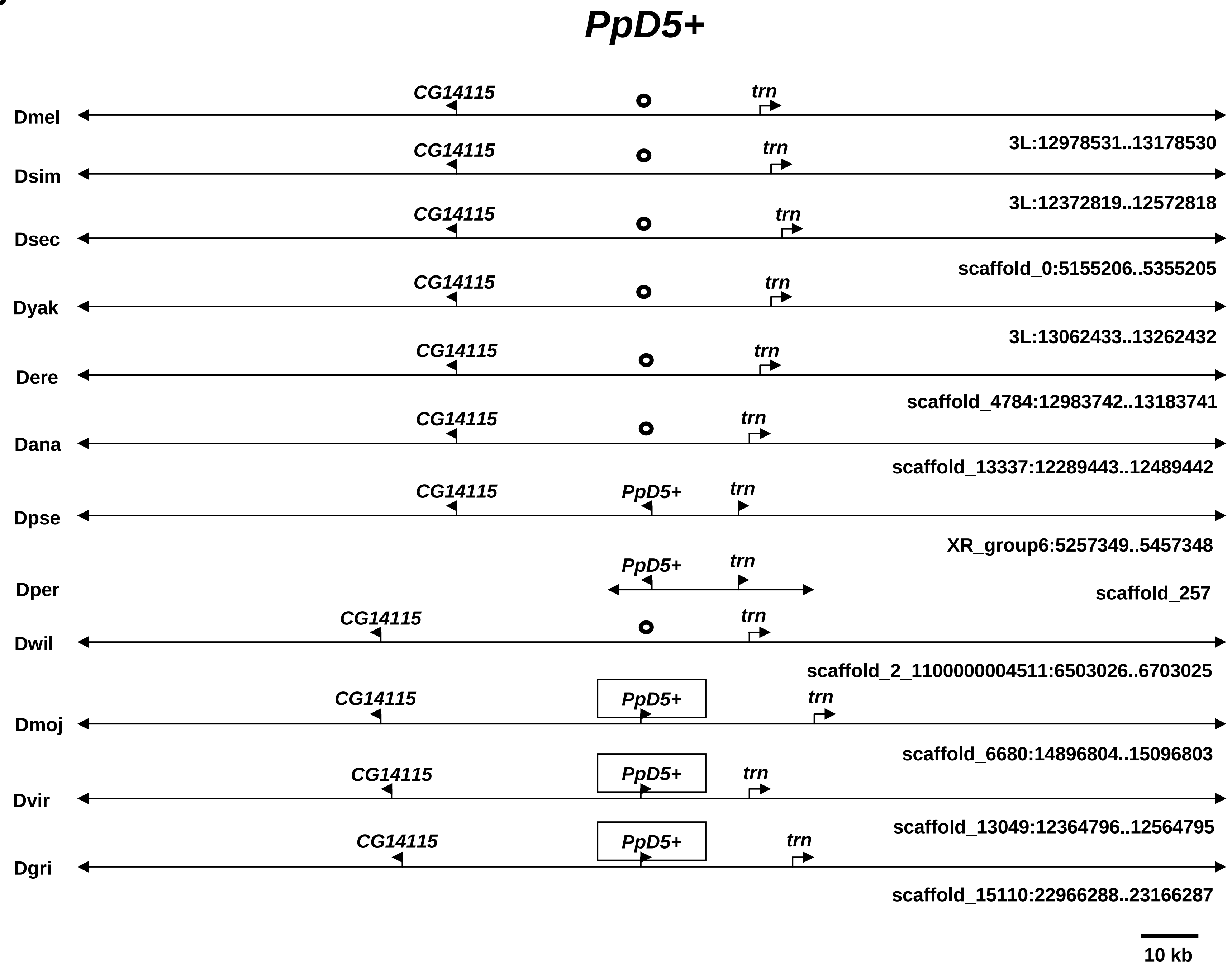

PpD5+
Dmel
CG14115
trn
3L:12978531..13178530
Dsim
CG14115
trn
3L:12372819..12572818
Dsec
CG14115
trn
scaffold_0:5155206..5355205
Dyak
CG14115
trn
3L:13062433..13262432
Dere
CG14115
trn
scaffold_4784:12983742..13183741
Dana
CG14115
trn
scaffold_13337:12289443..12489442
Dpse
CG14115
PpD5+
trn
XR_group6:5257349..5457348
Dper
PpD5+
trn
scaffold_257
Dwil
CG14115
trn
scaffold_2_1100000004511:6503026..6703025
Dmoj
CG14115
PpD5+
trn
scaffold_6680:14896804..15096803
Dvir
CG14115
PpD5+
trn
scaffold_13049:12364796..12564795
Dgri
CG14115
PpD5+
trn
scaffold_15110:22966288..23166287
10 kb

Figure S4D

PpD5+

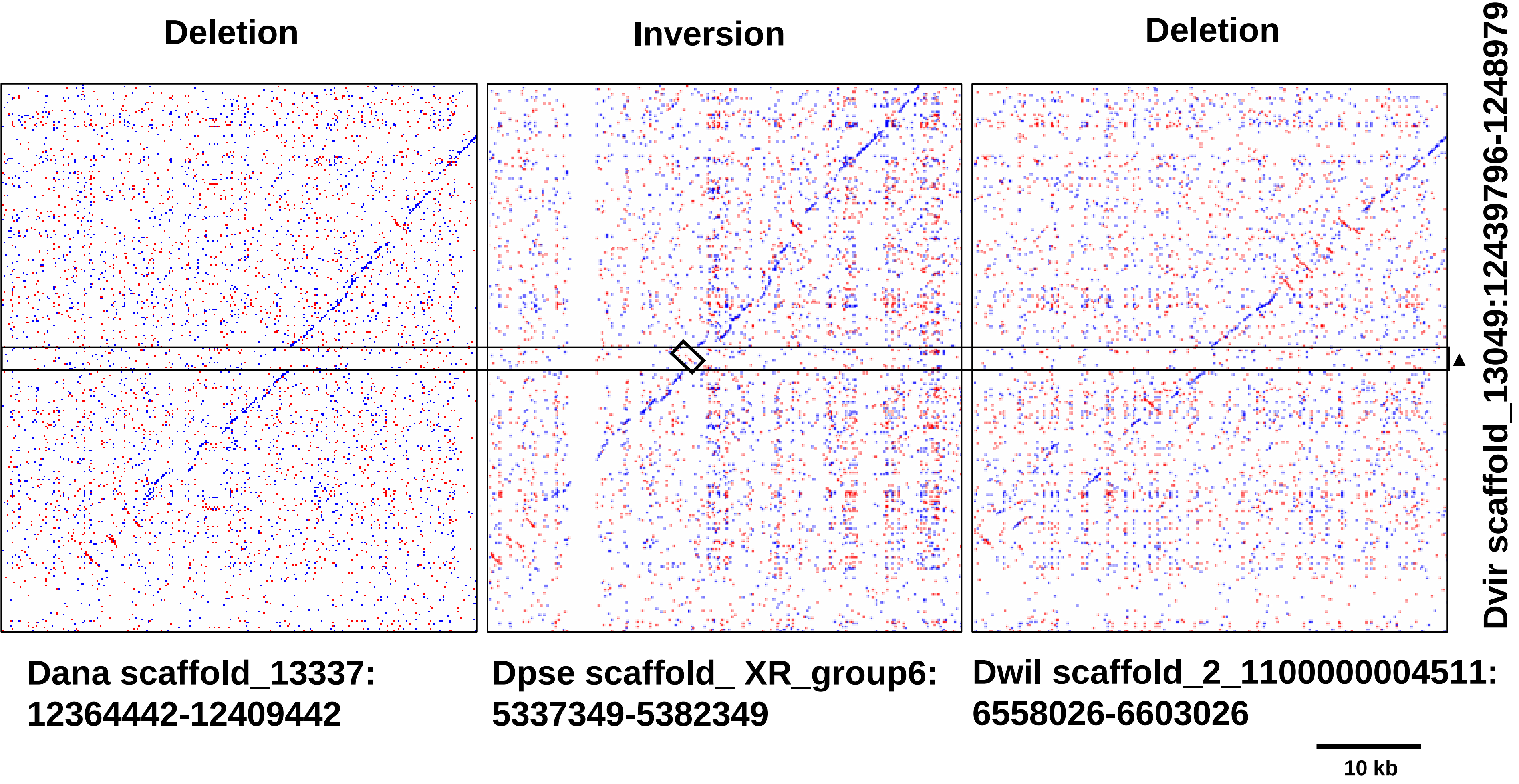

Figure S4E

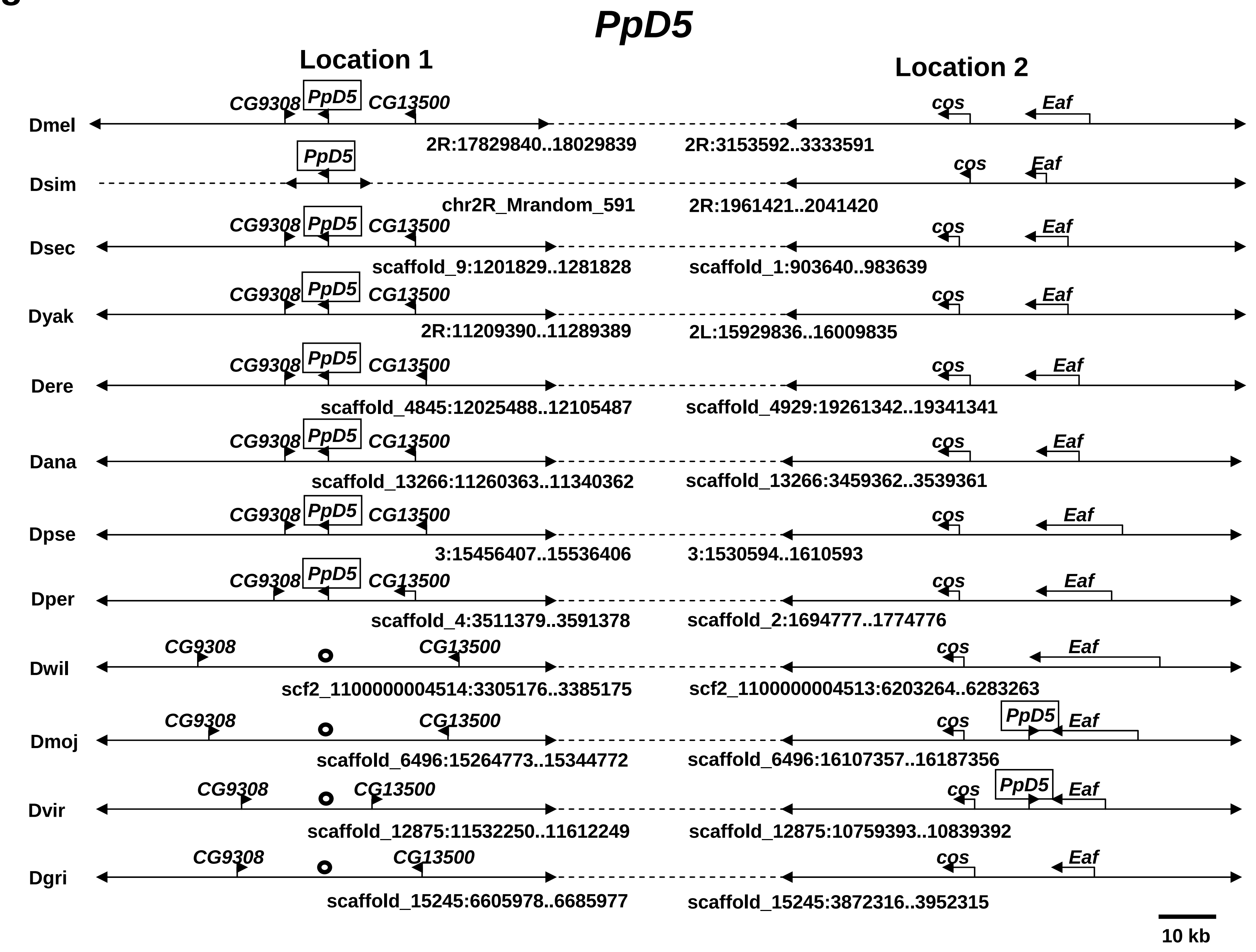

PpD5
Location 1
Location 2
Dmel
CG9308
PpD5
CG13500
cos
Eaf
2R:17829840..18029839
2R:3153592..3333591
Dsim
chr2R_Mrandom_591
2R:1961421..2041420
Dsec
scaffold_9:1201829..1281828
scaffold_1:903640..983639
Dyak
2R:11209390..11289389
2L:15929836..16009835
Dere
scaffold_4845:12025488..12105487
scaffold_4929:19261342..19341341
Dana
scaffold_13266:11260363..11340362
scaffold_13266:3459362..3539361
Dpse
3:15456407..15536406
3:1530594..1610593
Dper
scaffold_4:3511379..3591378
scaffold_2:1694777..1774776
Dwil
scf2_1100000004514:3305176..3385175
scf2_1100000004513:6203264..6283263
Dmoj
scaffold_6496:15264773..15344772
scaffold_6496:16107357..16187356
Dvir
scaffold_12875:11532250..11612249
scaffold_12875:10759393..10839392
Dgri
scaffold_15245:6605978..6685977
scaffold_15245:3872316..3952315
10 kb

Figure S4F

PpD5

Gene Degradation in Location 1

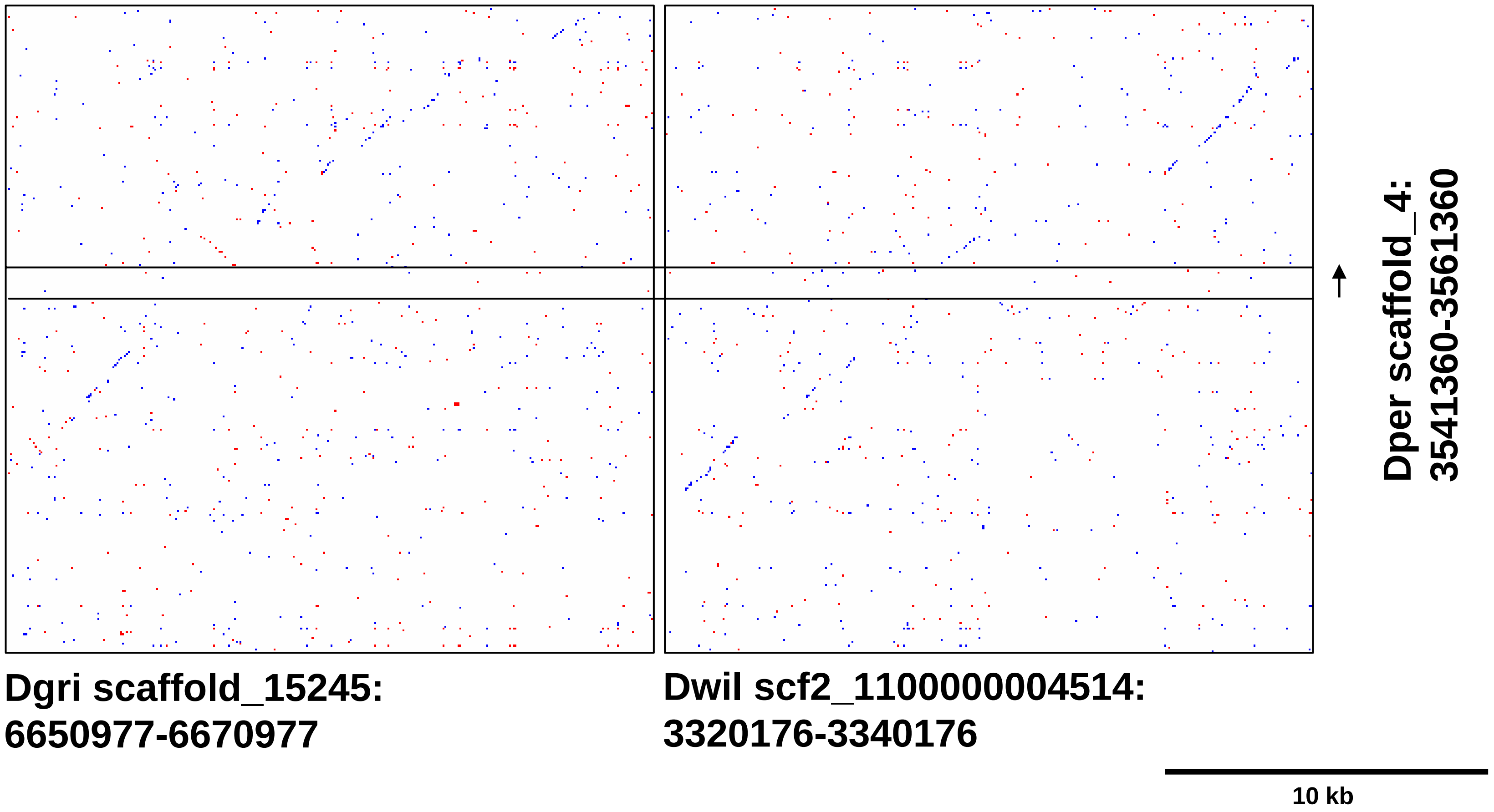

Figure S4G

PpD5

Gene Insertion in Location 2

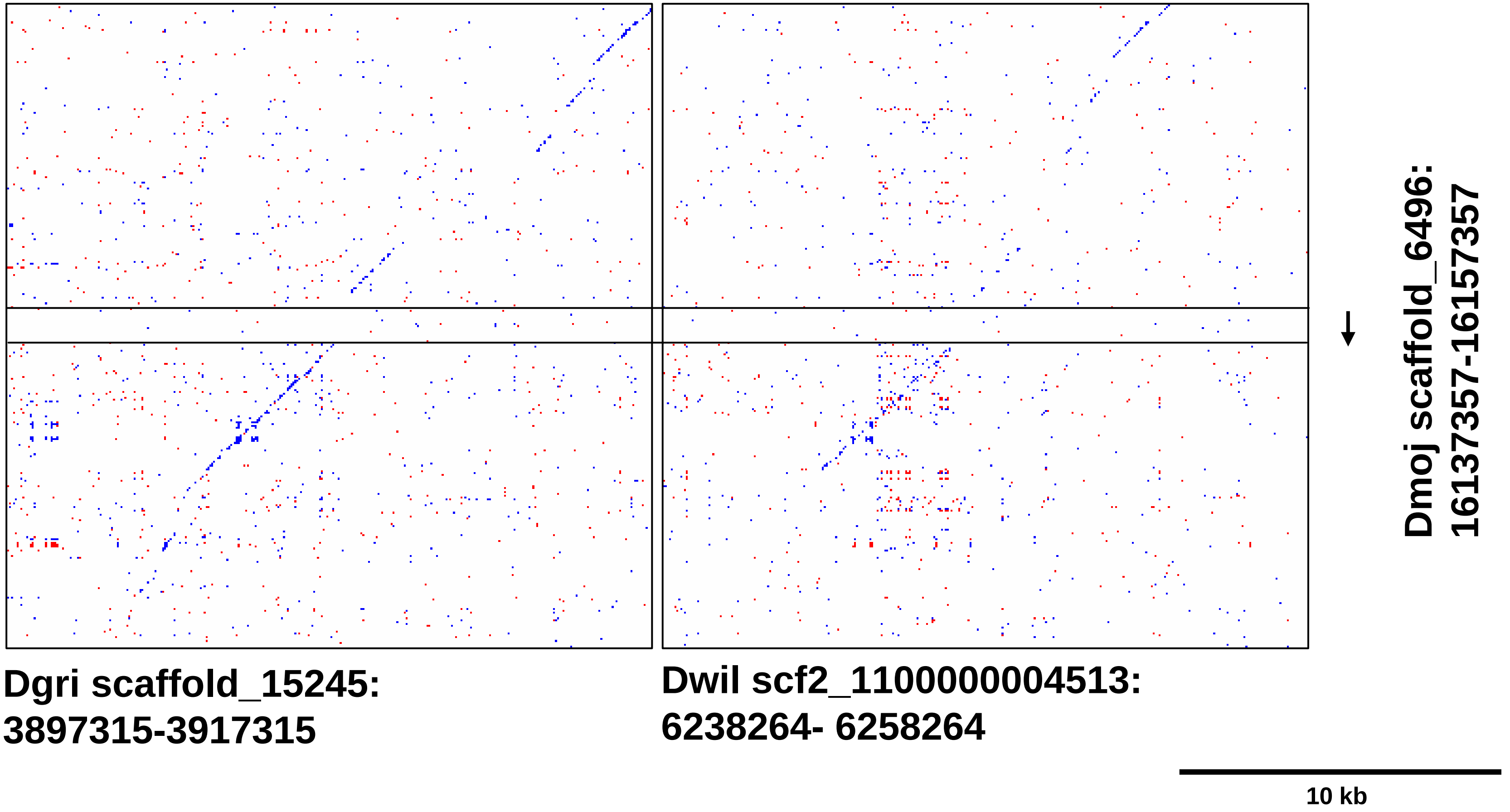

Supplement: Figure S4 — Analysis of specific PPP gene movements in Drosophilidae . The synteny of PpY-55A, PpD5+, and PpD5 genes are shown in panels (A), (C), and (E). Homologous chromosomal regions of about 200 kbp are represented by double headed arrows. Abridged species names are on the left, chromosome/scaffold identifications and ranges are either on the right side or in the middle of the panels. Broken lines indicate large DNA segments that are situated between the two depicted areas. Arrows show the direction and size of landmark genes, o labels the expected position of a missing gene. The names of the intronless genes are boxed. Dot plots compare homologous chromosomal regions containing the PpY-55A (B), PpD5+ (D), and PpD5 (F, G) genes from selected Drosophila species. In (B) broken lines delimit two large inversions, which are circled in the plot. The inverted PpY-55A gene is boxed. In (D) a small arrow at the right side of the plots shows the size and direction of the PpD5+ gene in D. virilis. This gene is expected to occur in the areas between the two horizontal lines. The inverted PpD5+ gene is boxed in D. pseudoobscura. The corresponding gene region was deleted from D. ananassae and D. willistoni. In (F) the arrow shows the size and direction of the PpD5 gene in D. persimilis. This gene is expected to occur in the areas between the two horizontal lines, but can not be recognized in D. grimshawi and D. willistoni because its sequence has been degraded. Panel (G) demonstrates that the chromosomal region in question is missing from D. grimshawi and D. willistoni indicating that PpD5 was inserted into this location in D. mojavensis. The scale indicates 10 kbp in all panels. (PDF) [file pone.0022218.s004.pdf]
