# Supplementary material for: Molecular Evolution of Phosphoprotein Phosphatases in Drosophila
Source: PLoS One. 2011 Jul 15;6(7):e22218. doi: 10.1371/journal.pone.0022218 (PMC3137614; doi:10.1371/journal.pone.0022218)
Supplement: Figure S5 — Chromosomal localization of PPP genes in Muller elements A (A), B (B), C (C), D (D), and E (E) of 12 Drosophila species. Abridged species names are given at the right side of the panels. Horizontal open bars represent continuous chromosomes or chromosome arms and a gap indicates a missing DNA sequence. A vertical line shows the localization, and the arrow tell the direction (left to right is 5′ to 3′ in the upper strand) of a given gene. The scale bar is 2 Mbp in all cases. (PPT) [file pone.0022218.s005.ppt]

## Slide 1
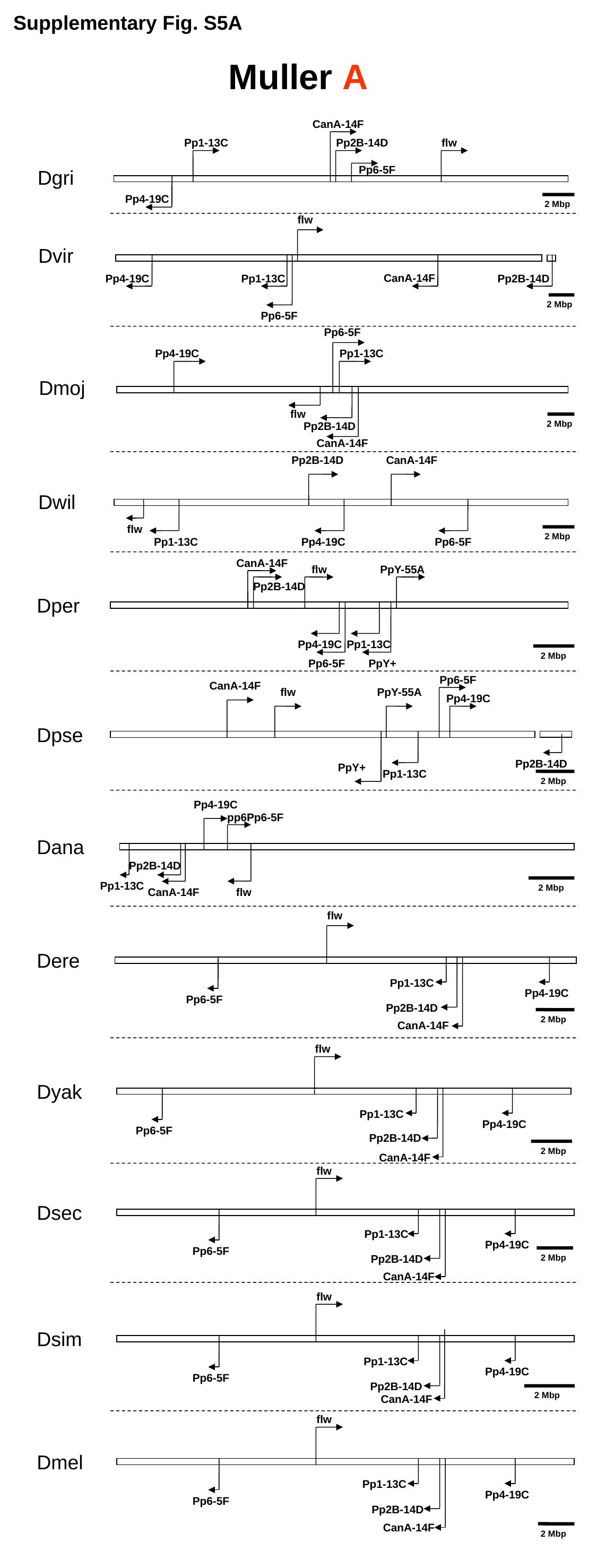

Supplementary Fig. S5A
Muller A
CanA-14F
Pp1-13C
Pp2B-14D
flw
Pp6-5F
Dgri
Pp4-19C
2 Mbp
flw
Dvir
CanA-14F
Pp4-19C
Pp1-13C
Pp2B-14D
2 Mbp
Pp6-5F
Pp6-5F
Pp4-19C
Pp1-13C
Dmoj
flw
2 Mbp
Pp2B-14D
CanA-14F
Pp2B-14D
CanA-14F
flw
Pp1-13C
Pp4-19C
Pp6-5F
Dwil
2 Mbp
CanA-14F
flw
PpY-55A
Pp2B-14D
Dper
Pp1-13C
Pp4-19C
2 Mbp
Pp6-5F
PpY+
Pp6-5F
CanA-14F
flw
PpY-55A
Pp4-19C
Dpse
Pp2B-14D
PpY+
Pp1-13C
2 Mbp
Pp4-19C
pp6Pp6-5F
Dana
Pp2B-14D
Pp1-13C
2 Mbp
CanA-14F
flw
flw
Dere
Pp1-13C
Pp4-19C
Pp6-5F
Pp2B-14D
2 Mbp
CanA-14F
flw
Dyak
Pp1-13C
Pp4-19C
Pp6-5F
Pp2B-14D
2 Mbp
CanA-14F
flw
Dsec
Pp1-13C
Pp4-19C
Pp6-5F
Pp2B-14D
2 Mbp
CanA-14F
flw
Dsim
Pp1-13C
Pp4-19C
Pp6-5F
Pp2B-14D
2 Mbp
CanA-14F
flw
Dmel
Pp1-13C
Pp4-19C
Pp6-5F
Pp2B-14D
CanA-14F
2 Mbp

## Slide 2
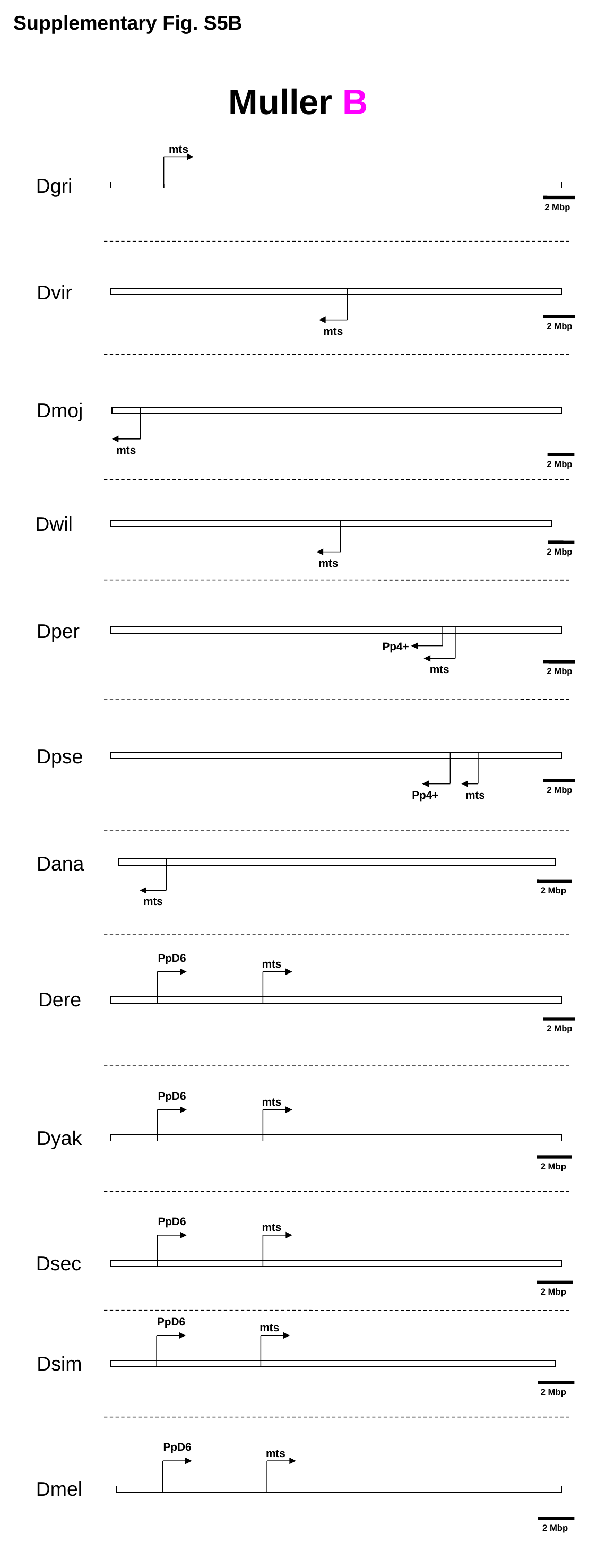

Supplementary Fig. S5B
Muller B
mts
Dgri
2 Mbp
Dvir
mts
2 Mbp
Dmoj
mts
2 Mbp
Dwil
mts
2 Mbp
Dper
Pp4+
mts
2 Mbp
Dpse
Pp4+
mts
2 Mbp
Dana
mts
2 Mbp
PpD6
mts
Dere
2 Mbp
PpD6
mts
Dyak
2 Mbp
PpD6
mts
Dsec
2 Mbp
PpD6
mts
Dsim
2 Mbp
PpD6
mts
Dmel
2 Mbp

## Slide 3
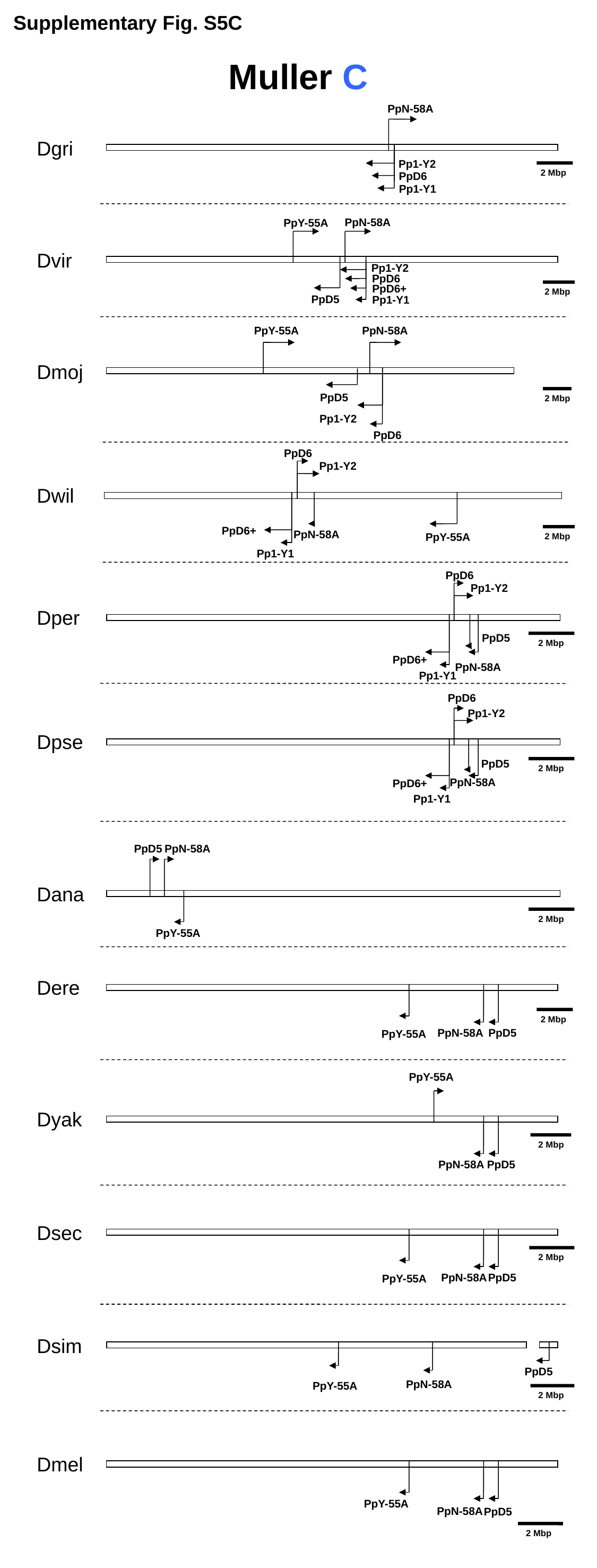

Supplementary Fig. S5C
Muller C
PpN-58A
Dgri
Pp1-Y2
2 Mbp
PpD6
Pp1-Y1
PpN-58A
PpY-55A
Dvir
Pp1-Y2
PpD6
PpD6+
2 Mbp
PpD5
Pp1-Y1
PpY-55A
PpN-58A
Dmoj
PpD5
2 Mbp
Pp1-Y2
PpD6
PpD6
Pp1-Y2
PpD6+
PpN-58A
PpY-55A
Pp1-Y1
Dwil
2 Mbp
PpD6
Pp1-Y2
Dper
PpD5
2 Mbp
PpD6+
PpN-58A
Pp1-Y1
PpD6
Pp1-Y2
Dpse
PpD5
2 Mbp
PpN-58A
PpD6+
Pp1-Y1
PpD5
PpN-58A
PpY-55A
Dana
2 Mbp
Dere
2 Mbp
PpN-58A
PpD5
PpY-55A
PpY-55A
Dyak
2 Mbp
PpN-58A
PpD5
Dsec
2 Mbp
PpN-58A
PpD5
PpY-55A
Dsim
PpD5
PpN-58A
PpY-55A
2 Mbp
Dmel
PpY-55A
PpN-58A
PpD5
2 Mbp

## Slide 4
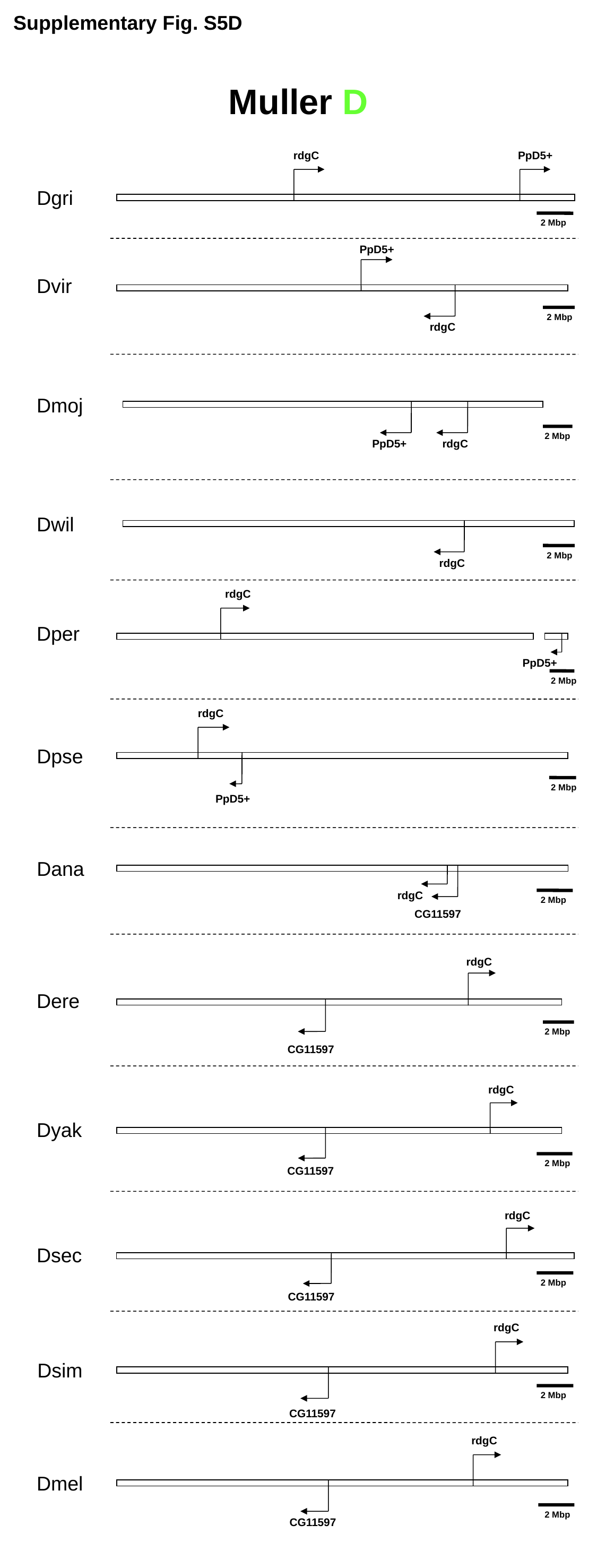

Supplementary Fig. S5D
Muller D
rdgC
PpD5+
Dgri
2 Mbp
PpD5+
Dvir
2 Mbp
rdgC
Dmoj
2 Mbp
PpD5+
rdgC
Dwil
rdgC
2 Mbp
rdgC
Dper
PpD5+
2 Mbp
rdgC
Dpse
2 Mbp
PpD5+
Dana
rdgC
2 Mbp
CG11597
rdgC
Dere
2 Mbp
CG11597
rdgC
Dyak
2 Mbp
CG11597
rdgC
Dsec
2 Mbp
CG11597
rdgC
Dsim
2 Mbp
CG11597
rdgC
Dmel
2 Mbp
CG11597

## Slide 5
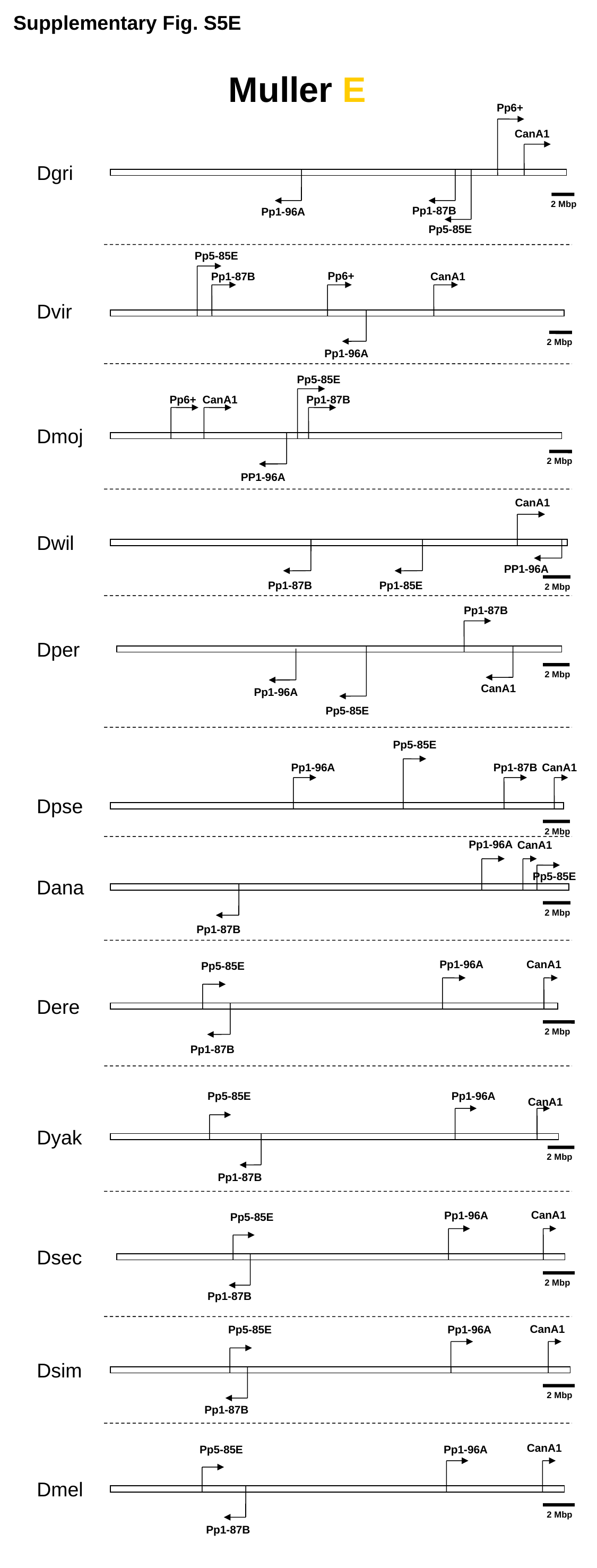

Supplementary Fig. S5E
Muller E
Pp6+
CanA1
Dgri
2 Mbp
Pp1-87B
Pp1-96A
Pp5-85E
Pp5-85E
Pp6+
Pp1-87B
CanA1
Dvir
2 Mbp
Pp1-96A
Pp5-85E
Pp6+
CanA1
Pp1-87B
Dmoj
2 Mbp
PP1-96A
CanA1
Dwil
PP1-96A
Pp1-87B
Pp1-85E
2 Mbp
Pp1-87B
Dper
2 Mbp
CanA1
Pp1-96A
Pp5-85E
Pp5-85E
Pp1-96A
Pp1-87B
CanA1
Dpse
2 Mbp
Pp1-96A
CanA1
Pp5-85E
Dana
2 Mbp
Pp1-87B
Pp1-96A
CanA1
Pp5-85E
Dere
2 Mbp
Pp1-87B
Pp5-85E
Pp1-96A
CanA1
Dyak
2 Mbp
Pp1-87B
CanA1
Pp1-96A
Pp5-85E
Dsec
2 Mbp
Pp1-87B
CanA1
Pp5-85E
Pp1-96A
Dsim
2 Mbp
Pp1-87B
CanA1
Pp1-96A
Pp5-85E
Dmel
2 Mbp
Pp1-87B
