# Supplementary material for: Molecular Evolution of Phosphoprotein Phosphatases in Drosophila
Source: PLoS One. 2011 Jul 15;6(7):e22218. doi: 10.1371/journal.pone.0022218 (PMC3137614; doi:10.1371/journal.pone.0022218)
Supplement: Table S1 — The sequences of oligonucleotides and the experimental conditions used for PCR or RT-PCR are summarized in three sections. (A) Oligonucleotide primers and conditions used for the detection of Drosophila PPP transcripts by RT-PCR. (B) Oligonucleotide primers and conditions used for the amplification and sequencing of Drosophila PPP genes. (C) Oligonucleotide primers and conditions used for the amplification of Drosophila RpL23 in control experiments. (DOC) [file pone.0022218.s006.doc]

**Table S1. Oligonucleotide primers and experimental conditions used in PCR and RT-PCR.**

**(A)** Oligonucleotide primers and conditions used for the detection of *Drosophila* PPP transcripts by RT-PCR.

| Target | Species | Forward primer | Reverse primer | Annealing  temperature(°C) | Size of the  amplicon  (bp) |
| --- | --- | --- | --- | --- | --- |
| ***Pp1-13C*** | Dmel | CAATTTGGAGAGCATAATTTCC | CAACTTGATGGTATATCTGCG | 60 | 424 |
|  | Dana | CGAGTCGAACTATCTCTTCC | GAAGGAGCACATGAGAGTG | 60 | 631 |
|  | Dpse | GGTTGTTGGCAAATTCCTC | TCAGGATCTGGAACGAGC | 60 | 200 |
|  | Dwil | CAGAGGTGCTTAATCTGGATAG | ATAGTTCGATTCTGGTGGATAG | 60 | 231 |
|  | Dvir | AGATCAAACGGATTATGCGG | TCGTAACCATCCTCGACAAC | 60 | 214 |
| ***PpD5+*** | Dpse | CGAATATGAGGTGTTCAATGCC | AGGTAATTATCAGATTGGCGTCC | 60 | 869/928^a^ |
|  | Dvir | AGCTGGCTAGTTGTGATAATCC | CCAGAGTAGGTCGCATACAAG | 60 | 569 |
| ***PpD6****+* | Dpse | ACATCGAGGGCATCATAAGAAC | AGATGAGATCGAAGTCATTCCG | 60 | 702 |
|  | Dwil | TTGTTAGCCGTCTGTTATCTGG | TCCTGACTTTGACTACCTTGTG | 60 | 842 |
|  | Dvir | GCGACGTTATTCTACGAAACTC | CGTTTAACACCTGATATGACCG | 60 | 522 |
| ***PpY****+* | Dpse | CGATGCTCCTGGAGCTGGAT | GAAGCTGACGACGAGGTCGC | 60 | 731 |
| ***Pp4-19C*** | Dmel | GACAGATCGAGCAACTGAAG | TTAGAGAAAGTAGTCCGCCTG | 60 | 899/963^a^ |
|  | Dana | GATCGCATAACACTCATCCG | TACTCGTTCAGTTCCAGAATGG | 60 | 518/603^a^ |
|  | Dpse | CATCAAAGAGAATGAGGTGAAG | GAAGATGACAAAGTCACGATG | 60 | 802/877^a^ |
|  | Dwil | CCTGCGTAAATATGGATCAAC | TAAAGAAAGTAATCCGCCTGAG | 60 | 534/608^a^ |
|  | Dvir | GATCGCATAACGCTGATACG | GTGGCTTCTTTGAGGGTATG | 60 | 589/683^a^ |
| ***Pp4****+* | Dpse | ATCATCGACGGCAAGATATTC | CGGATGGAAGATCACAAAGTC | 60 | 402 |
| ***PpV/Pp6-5F*** | Dmel | GAAGACGTGAAGAAATGCAAG | AGGTTCAGATTATTGATCGCC | 60 | 665/841^a^ |
|  | Dana | GGTAGTCAAGGAGTGCAAATAC | AACATTATGACCAAATAGCCAG | 60 | 628/748^a^ |
|  | Dpse | ATGGATAGAGATTGTGAAGGAG | AGGAAATAGGGTGTCGTATTC | 60 | 891/1054^a^ |
|  | Dwil | CAACATACAGCCAGTGAGC | TACGGTATCTCACCATTTCG | 64 | 444/1391^a^ |
|  | Dvir | TTGGAGGAGACAAACATACAG | GTATCGAAGCTGAGTATGGC | 64 | 722/1105^a^ |
| ***Pp6****+* | Dvir | TGAGATAGTGGAGAAGTGTCAG | GATAGTGAGCAGATCGAAGAC | 60 | 424 |

^a^In these species the phosphatase genes contain short introns. The sizes of the amplicons from both cDNA/genomic DNA are given.

**Table S1 (continued)**

**(B)** Oligonucleotide primers and conditions used for the amplification and sequencing of *Drosophila* PPP genes.

| Target | Species | Forward primer | Reverse primer | Annealing  temperature(°C) | Size of the  amplicon (bp) |
| --- | --- | --- | --- | --- | --- |
| ***Pp1-Y1*** | Dmel, Dsim, Dsec,  Dyak, Dere | TAAAGTGCGTTATCCGACGAGC | AGCACCGGCATTATCAAACTCC | 62 | 514 |
| ***Pp1-Y2*** | Dmel, Dsim, Dsec, | TGCGAATTATTGACACTCGCC | ATGTGCACGGCAAATGAGATC | 62 | 695 |
| ***PpD5*** | Dsim, Dper | GACCTGGTGAGGATATTCAAGAAG | CTGGCGGTTAGCAAAGAACTC | 60 | 576 |
| ***PpD6*** | Dere, Dpse | AACTTTTCCTGGACGAGCCCATG | TAGTTGGGTGCCGAGAAGACGG | 69 | 717 |
| ***PpD6*** | Dana | CGAGCCAGCAAAGTAACC | GGAGCGGAAAAAACAGTC | 60 | 1345 |
| ***PPN-58A*** | Dper | CAGAGGAGCGGAATGGAG | GGCATATCAGCGTGAAGTG | 60 | 727 |
| ***PPY-55A*** | Dper | CGTGGCTAACCGTATCTTCTG | GTCGCTGACACACATCACTG | 60 | 370 |
| ***CanA1*** | Dper | ACATTCACGGACAGTTCTTC | AATCCTTTCCTCCAGACAAC | 54 | 2499 |
| ***CanA-14F*** | Dmel, Dsim, Dsec | ACAACACACACGACAACAAG | GGCTGACAGTGATTTCTGG | 67 | 1613 |

**(C)** Oligonucleotide primers and conditions used for the amplification of *Drosophila* *RpL23* in control experiments.

| Target | Species | Forward primer | Reverse primer | Annealing  temperature(°C) | Size of the  genomic fragment  (bp) | Size of the  cDNA  (bp) |
| --- | --- | --- | --- | --- | --- | --- |
| ***RpL23*** | Dmel | GTGATGAACTGTGCCGACAA | CCTTCATTTCGCCCTTGTTG | 50 | 854 | 268 |
|  | Dana | GTGATGAACTGTGCCGACAA | CCTTCATTTCGCCCTTGTTG | 50 | 764 | 268 |
|  | Dpse | GTGATGAACTGTGCCGACAA | CCTTCATTTCGCCCTTGTTG | 50 | 522 | 268 |
|  | Dwil | GTGATGAACTGTGCCGACAA | CCTTCATTTCGCCCTTGTTG | 50 | 414 | 268 |
|  | Dvir | GTGATGAACTGTGCCGACAA | CCTTCATTTCGCCCTTGTTG | 50 | 723 | 268 |
