# Supplementary material for: Molecular Evolution of Phosphoprotein Phosphatases in Drosophila
Source: PLoS One. 2011 Jul 15;6(7):e22218. doi: 10.1371/journal.pone.0022218 (PMC3137614; doi:10.1371/journal.pone.0022218)
Supplement: Table S3 — The G−C content in % is given for the coding regions of all Drosophila genes that changed location between Y chromosome and other (somatic or X) chromosomes. (DOC) [file pone.0022218.s008.doc]

**Table S3. The G-C content (%) of the coding regions in the *Drosophila* genes that changed location between Y chromosome and other (somatic or X) chromosomes.**

| **Species/Gene^a^** | ***PPr-Y*** | ***PRY*** | ***kl-5*** | ***ARY*** | ***CCY*** | ***WDY*** | ***Pp1-Y1*** | ***Pp1-Y2*** | ***PpD6*** |
| --- | --- | --- | --- | --- | --- | --- | --- | --- | --- |
| ***Dmel*** | **34.9** | **36.2** | **40.3** | **35.4** | **39.4** | **43.8** | **45.0** | **42.9** | **51.5** |
| ***Dyak*** | **34.6** | **37.4** | **40.9** | **36.1** | **39.1** | **43.1** | **42.2** | **43.8** | **52.0** |
| ***Dere*** | **34.7** | **35.2** | **40.9** | **36.2** | **39.6** | **43.2** | **41.7** | **43.3** | **52.0** |
| ***Dana*** | **32.9** | **35.2** | **38.4** | **38.1** | **38.0** | **40.8** | **42.9** | **44.3** | **46.1** |
| ***Dpse*** | **35.3** | **50.2^b^** | **53.1^c^** | **38.4** | **54.0** | **53.9** | **58.5** | **57.5** | **55.2** |
| ***Dwil*** | **34.8** | **36.3** | **42.0** | **37.8** | **39.1** | **43.7** | **42.3** | **44.4** | **43.1** |
| ***Dmoj*** | **35.2** | **37.8** | **37.2** | **50.5** | **47.6** | **50.9** | **x** | **57.2** | **53.2** |
| ***Dvir*** | **34.9** | **39.2** | **38.9** | **46.8** | **50.0** | **53.0** | **48.0** | **49.5** | **47.5** |
| ***Dgri*** | **x** | **37.6^b^** | **38.8** | **44.5** | **49.0** | **51.2** | **45.2** | **49.0** | **48.5** |

^a^ The genes in Y are shown in yellow background. The chromosomal localization of these genes was reported by Koerich et al. [31] and by Adam et al. [8]. Three of the localizations were revised in the present study (see b and c).

^b^ The *PRY* gene is in the XL chromosome (Muller element A^-^) of *D. pseudoobscura* while it is in the Y chromosome of *D. grimshawi.*

^c^ The kl-5 gene is in chromosome 2 (Muller element E) of *D. pseudoobscura.*

x stands for a lost or missing gene.
