# Supplementary material for: Molecular Evolution of Phosphoprotein Phosphatases in Drosophila
Source: PLoS One. 2011 Jul 15;6(7):e22218. doi: 10.1371/journal.pone.0022218 (PMC3137614; doi:10.1371/journal.pone.0022218)

Supplementary Fig. S1

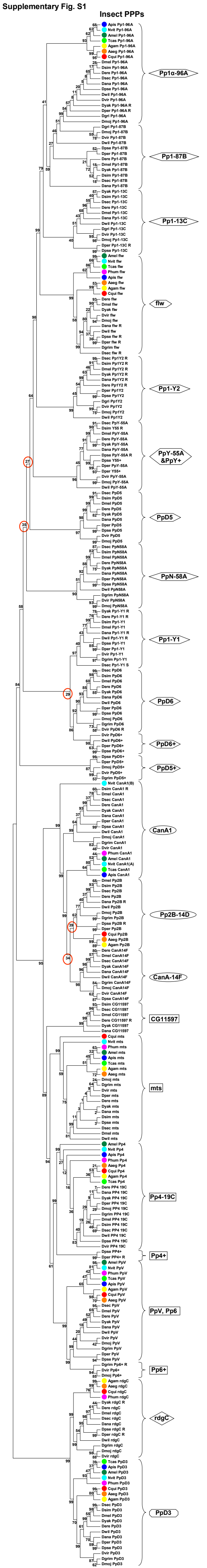

Supplement: Figure S1 — The protein identifications for 287 insect PPP catalytic subunits and the bootstrap values corresponding to Figure 2 are shown. The color codes of the proteins from non-Drosophila species are the same as in Figure 2 . Uncertain branching points are circled. (PDF) [file pone.0022218.s001.pdf]
